# The influence of socioeconomic status on the association between unhealthy lifestyle factors and adverse health outcomes: a systematic review

**DOI:** 10.12688/wellcomeopenres.18708.1

**Published:** 2023-02-03

**Authors:** Hamish M.E. Foster, Peter Polz, Jason M.R. Gill, Carlos Celis-Morales, Frances S. Mair, Catherine A. O'Donnell

**Affiliations:** 1 General Practice and Primary Care, School of Health and Wellbeing, College of Medical, Veterinary and Life Sciences, University of Glasgow, Glasgow, Scotland, G12 9LX, UK; 2 School of Cardiovascular and Metabolic Health, College of Medical, Veterinary and Life Sciences, University of Glasgow, Glasgow, Scoland, G12 8TA, UK

**Keywords:** Public Health, Epidemiology, Lifestyle, Health Behaviour, Healthcare Disparities, Socioeconomic Factors

## Abstract

**Background:** Combinations of lifestyle factors (LFs) and socioeconomic status (SES) are independently associated with cardiovascular disease (CVD), cancer, and mortality. Less advantaged SES groups may be disproportionately vulnerable to unhealthy LFs but interactions between LFs and SES remain poorly understood. This review aimed to synthesise the available evidence for whether and how SES modifies associations between combinations of LFs and adverse health outcomes.

**Methods:** Systematic review of studies that examine associations between combinations of >3 LFs and health outcomes and report data on SES influences on associations. Databases (PubMed/EMBASE/CINAHL), references, forward citations, and grey-literature were searched from inception to December 2021. Eligibility criteria were analyses of prospective adult cohorts that examined all-cause mortality or CVD or cancer mortality/incidence.

**Results:** Six studies (n=42,467–399,537; 46.5–56.8 years old; 54.6–59.3% women) of five cohorts were included. All examined all-cause mortality; three assessed CVD/cancer outcomes. Four studies observed multiplicative interactions between LFs and SES, but in opposing directions. Two studies tested for additive interactions; interactions were observed in one cohort (UK Biobank) and not in another (NHANES). All-cause mortality HRs (95% CIs) for unhealthy LFs ( *versus* healthy LFs) from the most advantaged SES groups ranged from 0.68 (0.32–1.45) to 4.17 (2.27–7.69). Equivalent estimates from the least advantaged ranged from 1.30 (1.13–1.50) to 4.00 (2.22–7.14). In 19 analyses (including sensitivity analyses) of joint associations between LFs, SES, and all-cause mortality, highest all-cause mortality was observed in the unhealthiest LF-least advantaged suggesting an additive effect.

**Conclusions:** Limited and heterogenous literature suggests that the influence of SES on associations between combinations of unhealthy LFs and adverse health could be additive but remains unclear. Additional prospective analyses would help clarify whether SES modifies associations between combinations of unhealthy LFs and health outcomes.

Registration: Protocol is registered with PROSPERO ( CRD42020172588; 25 June 2020).

## Introduction

Unhealthy lifestyle factors (LFs) ( *e.g.*, smoking, alcohol, poor diet, low physical activity (PA)) are key modifiable risk factors for non-communicable diseases (NCDs) and mortality ^1^. While single LFs have, by themselves, strong associations with NCDs and mortality, combinations of unhealthy LFs have stronger associations. Meta-analyses show that, compared with healthy LFs, combinations of at least three unhealthy LFs are associated with more than twice the risk of all-cause, cardiovascular disease (CVD), and cancer mortality, and CVD incidence ^2, 3^. Examining adverse health outcomes associated with combinations of LFs can help to capture ‘real life’ risks more completely as unhealthy LFs tend to cluster together - individuals with one unhealthy LF often have more than one ^4, 5^. And the impacts of one unhealthy LF may interact (additively or multiplicatively) with other unhealthy LFs ^6– 8^.

In addition to examining the associations between combinations of LFs and adverse health outcomes ( *e.g.*, all-cause, CVD, and cancer mortality, and CVD incidence), examining the effect of socioeconomic status (SES) on those associations can deepen understanding of the distribution of these lifestyle-related adverse health outcomes among populations. As with most health outcomes, all-cause, CVD, and cancer mortality, and CVD incidence all follow clear and long-recognised SES-health gradients where individuals of less advantaged SES ( *e.g.*, those with lower educational attainment, lower income, or who live in areas of higher deprivation) tend to have higher rates of both morbidity and mortality ^9, 10^. SES is a theoretical construct that differentiates sections of society by their means and access to resources ( *e.g.*, financial, educational, material) and by the ways in which they live ( *e.g.*, occupation type or class, housing type/conditions, neighbourhood/post code area) ^10^. The broad scope that SES encompasses means 1) there are numerous ways in which SES can be operationalised or measured ^11, 12^; and 2) there are numerous aspects of SES that could be expected to influence and have strong associations with both LFs and lifestyle-related adverse health outcomes ^13, 14^. For example, there is higher prevalence of unhealthy LFs in less advantaged SES groups and clustering of multiple unhealthy LFs in such population groups is often cited as an explanation for observed lifestyle-related adverse health inequalities ^4^. However, ‘differential exposure’ to unhealthy LFs only partially explains lifestyle-related health inequalities; higher prevalence of unhealthy LFs is estimated to account for 6–80% of SES related mortality inequalities ^4, 15– 18^.

Beyond differential exposure, further explanations for lifestyle-related health inequalities may involve interactions between LFs and SES; so-called ‘differential vulnerability’ ^17^. A study of over 300,000 UK Biobank (UKB) participants observed multiplicative interactions between a combination of unhealthy LFs and SES, where less advantaged SES groups had disproportionately higher lifestyle-related all-cause and CVD mortality ^19^. Similar interactions between lifestyle and SES have been observed for single LFs: smoking, alcohol, and PA ^20– 22^. A multiplicative interaction between LFs and SES supports a vulnerability hypothesis, where less advantaged groups are disproportionately vulnerable to the adverse effects of unhealthy LFs ^17, 20^. Whereas additive interactions, where the effects of a combination of unhealthy LFs and SES are added rather than multiplied ^23^, can also highlight vulnerable groups and inform policy or interventions ^24^. Mechanisms that explain differential lifestyle vulnerability are unclear but could include interactions with other factors associated with less advantaged SES ( *e.g.*, stress, reduced access to health care) or accelerated biological ageing *via* greater cumulative risks over the life-course ( *e.g.*, poorer childhood health or increased adverse childhood experiences) ^25– 27^.

### Aims

Understanding whether SES influences the association between combinations of unhealthy LFs and adverse health outcomes could help reduce excess risk in less advantaged populations by deepening understanding of how complex lifestyle risks vary across society and by identifying higher risk LF combinations. This could inform health policy, guide the development of interventions targeting more vulnerable groups, and support health care professionals managing multiple risk factors in their patient population. This systematic review aims to identify, describe, and synthesise the evidence for whether SES modifies associations between combinations of unhealthy LFs and adverse health outcomes. This review addresses the following research questions: Does SES modify the association between combinations of unhealthy LFs and adverse health outcomes? And if so, how?

An important caveat: ‘unhealthy lifestyle’ can imply unhealthy choices made freely by individuals, leading to potential stigma. However, resource scarcity and the wider socioeconomic environment experienced by those in less advantaged SES groups clearly influences choices, for example, by making healthier choices less likely ^28, 29^. Moreover, unhealthy lifestyle choices in the context of poverty or material deprivation may represent ‘optimal’ choices given wider socioeconomic influences that shape decision making and abstract future planning ^30, 31^. Nevertheless, the word lifestyle remains recognised in the context of modifiable behaviours and is therefore used here.

## Methods

### Search strategy and study selection

This review followed a protocol and was conducted in accordance with Preferred Reporting Items for Systematic Reviews and Meta-Analyses (PRISMA) guidelines ^32– 34^. The protocol is registered with a database of prospectively registered systematic reviews (PROSPERO CRD42020172588; 25 June 2020) ^35, 36^.

Search strategies were developed with a specialist university librarian and adapted for three databases: PubMed (RRID:SCR_004846), EMBASE (RRID:SCR_001650), and EBSCO CINAHL (RRID:SCR_022707) (S1-3 Tables, which can be found as *Extended data*) ^37^. The search strategy of a previous systematic review of combinations of LFs and type 2 diabetes served as a template and was adapted to include SES related terms ^38^. As per that previous review, this current review focusses on combinations of LFs, and therefore search terms relating to LFs included general terms like ‘lifestyle’ or ‘health behaviour’ rather than terms for individual LFs like ‘smoking’ or ‘alcohol’. Search terms also included terms for combinations of LFs ( *e.g.*, ‘combined’, ‘multiple’, ‘score’). Searches from inception (PubMed-1966; EMBASE-1947; CINAHL-1984) to 17 ^th^ December 2021 were supplemented by searches of references, forward citations, and grey literature ^36^.

### Eligibility criteria and screening

Inclusion criteria were developed using an adapted PICOS (population, intervention, comparator, outcome, study design) framework, with ‘I’ (intervention) replaced with ‘E’ (exposure) ^39^. Inclusion criteria:

1) Population: any general adult population. Studies of participants with an index condition were excluded.
2) Exposure - examination of two main exposures:
  i. combination of ≥3 LFs: studies that also included metabolic/intermediate factors ( *e.g.*, blood pressure/body mass index (BMI)) as part of their combination of LFs were included so long as the combination also included ≥3 ‘behavioural’ LFs ( *e.g.*, smoking/PA/diet).
  ii. SES: any SES measure (e.g., income/education/poverty-index).
3) Comparator: data for the influence of SES on associations between combinations of unhealthy LFs and adverse health.
4) Outcomes: at least one from: all-cause mortality, incidence and mortality from CVD or cancer.
5) Study design: prospective observational cohort. All types of analysis were included, and no study was excluded based on analysis method.

Exclusion criteria: not in English; abstracts/conference presentations only; ineligible design ( *e.g.*, review/case-control/cross-sectional/qualitative). Studies were uploaded to ‘ DistillerSR’ software (Version 2.38. DistillerSR Inc.; 2022. Accessed December 2021-February 2022; alternative software, Rayyan) and duplicates removed. Two reviewers (PP and HF/CO’D) screened titles and abstracts independently. Conflicts were resolved by discussion or included for full-text screening. Two reviewers (PP and HF) screened full-texts independently; conflicts resolved by discussion with a third reviewer (CO’D).

### Data extraction

Two reviewers (HF and PP/CO’D) extracted data independently using a piloted proforma (S4 Table, which can be found as *Extended data*) ^37^. After peer review, the proforma was adapted to include the distribution of type and number of unhealthy LFs among participants ^36^. Quality was measured using the Newcastle-Ottawa Scale for cohort studies (NOS) ^40^. The NOS was adapted to include assessments of confounder adjustment, sensitivity analysis, and missing data methodology (S5 Table, which can be found as *Extended data*) ^36, 37^. To compare study results, the following data from SES stratified analyses for each outcome was used to form our ‘main comparator’: 1) risk estimates for participants with the unhealthiest LF combination (using healthiest LF combination as reference) in the most advantaged SES group ( *e.g.*, highest education, highest ranking occupation) were compared with 2) equivalent estimates (unhealthiest *versus* reference healthiest LF combination) in the least advantaged SES group ( *e.g.*, lowest education, lowest ranking occupation). Studies frequently used more than two categories/quantiles of LF combinations, however only the estimates for the healthiest and unhealthiest categories were extracted. For example, for a study with a lifestyle score based on eight LFs, which study authors classified into five categories (scores 0–3, 4, 5, 6, and 7–8), the estimates for scores 0–3 and 7–8 were extracted. Estimates from SES stratified analyses were used for the main comparator because some studies did not report analyses examining combined influence of LF and SES using a single reference group ( *i.e.*, analyses comparing all groups to the group with the healthiest combination of LFs and in the most advantaged SES group). However, results for these analyses were also extracted as they provide information on the combined influence of SES and lifestyle. To make direct comparisons, estimates from studies where the unhealthiest group was the reference were transformed to make the ‘healthiest’ group the reference.

Meta-analysis was not appropriate due to the heterogeneity of included studies. Instead, results were reported and synthesised according to Synthesis Without Meta-analysis (SWiM) guidelines ^41^. In accordance with transparent reporting of the synthesis methodology, this review adhered to the following approach - study results were grouped by outcome and compared by: 1) main models evaluating influence of SES; 2) model adjustment; 3) additional models, including sensitivity analyses; 4) tests for interactions; and 5) results for our main comparator.

## Results

**Figure 1.**
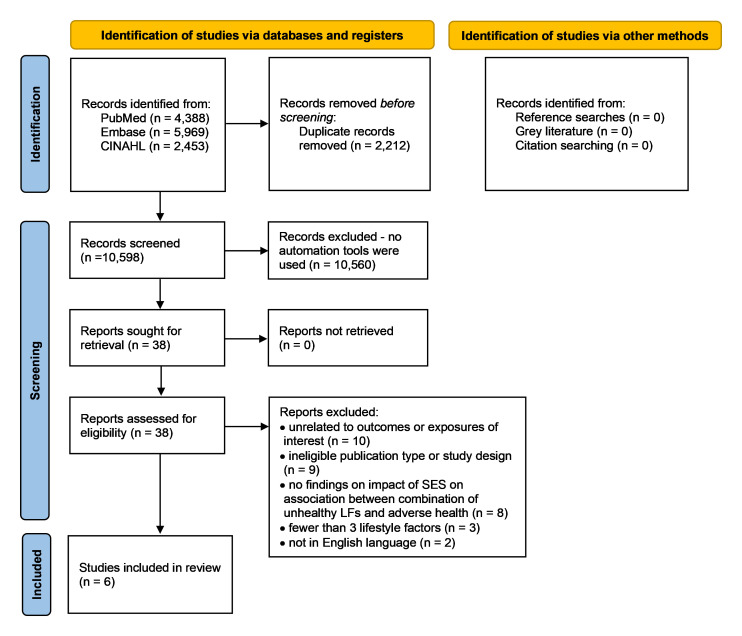
PRISMA flow chart of searches and screening results. CINAHL, Cumulative Index to Nursing and Allied Health Literature; SES, socioeconomic status; LFs, lifestyle factors.

Results of the searches and screening are shown in a PRISMA flowchart ( Figure 1).

### Study populations

Six studies of five cohorts were included in this review ( Table 1) ^19, 42– 46^. Two studies analysed the same USA-based cohort, The Southern Community Cohort Study (SCCS), but each study examined different LFs and SES exposures and therefore both were included ^42, 44^. Similarly, two studies analysed UKB and examined different exposure variables and outcomes ^19, 46^. The remaining cohorts analysed were The Japan Collaborative Cohort Study (JCCS) ^43^, the National Health Interview Survey (NHIS) ^45^, and US National Health and Nutrition Examination Survey (NHANES) ^46^. SCCS was designed to investigate ethnic inequalities in healthcare and 86% of participants were recruited from community health centres; JCCS, UKB, NHIS and NHANES are general population cohorts with NHIS and NHANES designed to be nationally representative ^47, 48^. Participants per study ranged from 42,467–399,537; mean age ranged from 46.5–56.8 years; and the proportion of women from 54.6–59.3%. Ethnic composition of populations analysed varied: SCCS cohort was 67% African American ^42, 44^; JCCS ethnicity was not reported, but likely predominantly Japanese ^43^; UKB was 95% White British ^19, 46^; NHIS ethnicity was not reported ^45^; NHANES was 73.6% White ^46^. Average follow-up time ranged from 4.3–19.3 years. In assessing the influence of SES on associations between combinations of unhealthy LFs and health outcomes, all studies examined all-cause mortality. In addition, three studies examined CVD mortality ^19, 43, 46^; two examined CVD incidence ^19, 46^; one examined heart disease mortality and incidence of myocardial infarction and stroke ^46^; two examined mortality from coronary heart disease (CHD), stroke, and cancer ^43, 46^; and one examined mortality from ‘non-CVD and non-cancer’ causes ^43^.

**Table 1.** Characteristics of included studies.

Author, year	Cohort Country Type N	Age (years) Women (%) Ethnicity	Follow up length	Lifestyle factors and definitions of unhealthy (source or justification for unhealthy definition) Categories for analysis *	SES measure Categories for analysis	Outcome
Andersen *et al.*, 2016 ^42^	Southern Community Cohort Study USA Prospective cohort designed to assess ethnic disparities in health outcomes, 86% recruited from community health centres 79,101 participants	Median age 51 (IQR 13) 59.3% women 67% African American	Max. 9 years (average not reported)	Self-reported at baseline Based on guidelines - i) current or former smoking (WHO) ii) alcohol intake >1 drink/d for women; >2 drinks/d for men (2010 Dietary Guidelines for Americans) iii) PA <150 min/wk moderate or <75 min/wk vigorous aerobic, or equivalent combination (2008 PA Guidelines for Americans) iv) sedentary time within 3 longest quartiles *i.e.*, >5.75 h/d (Avoid inactivity and limit sedentary behaviours; American Cancer Society) v) diet quality (HEI) in 3 lowest quartiles *i.e.*, <66.7 (USDA’s Center for Nutrition Policy and Promotion and previous publication) Five categories according to the number of lifestyle factor guidelines met (0, 1, 2, 3, ≥4).	Income self-reported at baseline Dichotomised as </≥ $15,000 p.a.	i) All-cause mortality *via* linkage to national registry
Eguchi *et al.*, 2017 ^43^	The Japan Collaborative Cohort Study Japan Prospective cohort of general population 42,647 participants	Mean age 56.8 (SD not reported) 56.8% women Likely to be mainly Japanese ethnicity	Median 19.3 years (IQR 11.6–20.8)	Self-reported at baseline i) current smoking ii) alcohol intake > 2 ‘gou’/d (>46 g ethanol/d) iii) PA: <0.5 h/d or <5 h/wk walking and/or in sports iv) sleep duration: <5.5 or >7.4 h/d v) BMI: <21 or >25 vi) fruit intake: <1x/d vii) fish intake: <1x/d viii) milk intake: <almost daily; Five categories: according to healthy lifestyle score with one point for each lifestyle factor threshold met (0–3, 4, 5, 6, 7–8)	Education level as age at last formal education self-reported at baseline Dichotomised as </≥ 16 years old	i) All-cause mortality ii) CVD mortality iii) CHD mortality iv) Stroke mortality v) Cancer mortality vi) Non-cancer and non-CVD mortality All *via* death certificate review
Andersen *et al.*, 2018 ^44^	Southern Community Cohort Study USA Prospective cohort designed to assess ethnic disparities in health outcomes, 86% recruited from community health centres 77,896 participants	Median age 51 (IQR 13) 57.1% women 66.1% African American	Median 8 years (IQR not reported)	Self-reported at baseline Based on guidelines - i) alcohol intake >1 drink/d for women, >2 drinks/d for men (2010 Dietary Guidelines for Americans) ii) PA <150 min/wk moderate or <75 min/wk vigorous aerobic, or equivalent combination (2008 PA Guidelines for Americans) iii) sedentary time within 3 longest quartiles *i.e.*, >6.5 h/d (Avoid inactivity and limit sedentary behaviours; American Cancer Society) iv) diet quality (HEI) in 3 lowest quartiles *i.e.*, <65.5 (USDA’s Center for Nutrition Policy and Promotion) Four categories: according to number of guidelines met (0, 1, 2, 3–4)	Neighborhood deprivation index (NDI): 2000 U.S. Census data linked to participant’s residential address incorporating education, employment, housing, occupation, and poverty Quartiles	i) All-cause mortality *via* linkage to national registry
Foster *et al.*, 2018 ^19^	UKB UK Prospective cohort of general population 328,594	Mean age 55.6 (SD 8.1) 54.6% women 95% White	Mean 4.9 years (SD 0.83, range 3.3–7.9) for all-cause and CVD mortality, 4.1 years (0.81 SD; range 2.4–7.0) for CVD incidence	Self-reported at baseline; Based on UK guidelines where available: i) current smoking ii) alcohol intake daily or almost daily iii) PA <150 min/wk moderate or <75 min/wk vigorous iv) TV viewing time ≥4 h/d v) sleep duration <7 or >9 h/d vi) fruits and vegetables <400g/d vii) oily fish <1 portion/wk viii) red meat >3 portions/wk ix) processed meat >1 portion/wk Three categories: according to lifestyle risk score with one point for each unhealthy definition met (0–2, 3–5, 6–9)	Townsend deprivation index: national census data incorporating car ownership, household overcrowding, owner occupation, and unemployment aggregated for and linked to participant postcode of residence Quintiles Secondary SES measures for sensitivity analyses: i) Household income (£p.a.) self-reported at recruitment Five categories: >100,000; 52,000-100,000; 30,000-51,999; 18,000-29,999; <18,000 ii) Educational attainment self-reported at recruitment Five categories: College/University degree; A levels or equivalent; O levels or equivalent; CSEs or equivalent; none of the above	i) All-cause mortality ii) CVD mortality iii) CVD incidence All *via* linkage to national registries
Choi *et al.*, 2022 ^45^	National Health Interview Survey USA Prospective cohort of general population aged ≥30 years 189,087	Age ≥30 (average not reported) Proportion female not reported Ethnicity not reported	Mean 12.7 years	Self-reported at baseline: i) current smoker and ex-smokers who quit <20 years ago ii) weekly alcohol intake >14 drinks for men, >7 drinks for women (2016 NIAAA guidelines), or >5 drinks/d at least monthly iii) PA <150 min/wk moderate or <75 min/wk vigorous and/or strengthening activities <2 d/wk (2008 Physical Activity Guidelines for Adults) iv) BMI <18.5 or ≥35 Five categories according to the number of lifestyle factors: 0, 1, 2, 3, or 4	Household income as a ratio of family income to federal poverty level Dichotomised as < or ≥200% of federal poverty level	i) All-cause mortality via linkage to national registry
Zhang *et al.*, 2021 ^46^	US National Health and Nutrition Examination Survey (NHANES) USA Prospective cohort of general population 44,462 & UKB UK Prospective cohort of general population 399,537	NHANES: Mean age 46.5 51.3% women 73.6% White UKB: Mean age 56.1 52.5% women 95.6% White	NHANES: Mean 11.2 years UKB: Mean 11.0 years for all-cause mortality, 8.8 years for CVD incidence	Self-reported at baseline; i) smoked >100 cigarettes in lifetime ii) daily alcohol intake >1 drink for women, 2 drinks for men (National guidelines for USA and UK) iii) leisure time physical activity at level of lower two thirds of study participants iv) diet quality (HEI) at level of lower 2/5 ^th^ of participants for US NHANES (2015-20 Dietary Guidelines for Americans & 1992 food guide from US Department of Agriculture); meeting 5/10 diet recommendations for UKB (evidence-based recommendations) Three categories according to number of lifestyle factors (score): 0–1, 2, 3–4 Sensitivity analyses included: a weighted lifestyle score to account for differing magnitude of associations between each LF and outcomes; and a combination of LFs that included BMI outwith 18.5–24.9.	NHANES: i) family poverty to income ratio: low (≤1); middle (1-4); and high (≥4) ii) educational attainment: less than high school diploma; high school graduate or equivalent; and college or above iii) occupation (US socioeconomic index): upper (index ≥50); lower (index <50); and unemployment iv) health insurance: private; public only; none Variables i)-iv) were self-reported at recruitment and combined *via* latent class analysis to generate 3 latent classes/categories of low, medium, and high SES UKB: i) income (£p.a.): >100,000; 52,000-100,000; 30,000-51,999; 18,000-29,999; <18,000 ii) educational attainment: College/University degree; A levels or equivalent; O levels or equivalent; CSEs or equivalent; NVQ, HND, HNC, or equivalent; other professional qualifications; none of the above iii) employment: employed (including self-employed, retired, unpaid/voluntary work, full/part time students); unemployed Variables i)-iii) were self-reported at recruitment and combined *via* latent class analysis to generate 3 latent classes/categories of low, medium, and high SES Secondary SES measures in sensitivity analyses included: each SES factor individually; Townsend index (UKB only)	i) All-cause mortality ii) CVD mortality iii) CVD incidence iv) heart disease mortality, NHANES only v) coronary heart disease mortality, UKB only vi) stroke mortality, UKB only vii) myocardial infarction incidence, UKB only vii) stroke incidence, UKB only All *via* linkage to national registries

N, number of participants included in analysis; *Categories for analysis shows the number of categories used by study authors to analyse the associations between the combination of lifestyle factors and health outcome ( *e.g.*, a study of five lifestyle factors, with possible scores of 0 to 5, could be analysed using the score categories of 0, 1, 2, 3, and ≥4; *i.e.*, with scores 4 and 5 grouped together); Outcomes, adverse health outcome used to assess interaction between lifestyle and SES (some studies reported additional health outcomes but these were not used to assess interaction); SES, socioeconomic status; IQR, Interquartile range; PA, physical activity; BMI, body mass index (kg/m ^2^); WHO, World Health Organisation; d, day; wk, week; h, hours; min, minutes; $, US dollars; pa per annum; £, British pounds; TV, television; HEI, Healthy Eating Index, which measures adherence to the Dietary Guidelines for Americans. HEI is based on 12 dietary components: total fruits; whole fruits; total vegetables; greens and beans; whole and refined grains; dairy; total protein foods; seafood and plant proteins; fatty acids; sodium; and calories from solid fats, alcohol, and added sugars (range 0–100; higher values indicate healthier diet); CVD, cardiovascular disease; CHD, coronary heart disease; UKB, UK Biobank; NIAAA, National Institute on Alcohol Abuse and Alcoholism; A level, General Certificate of Education Advanced Level; O-level, General Certificate of Education Ordinary Level; CSE, Certificate of Secondary Education; NVQ, National Vocational Qualification; HND/HNC, Higher National Diploma/Certificate.

### Combinations of unhealthy lifestyle factors

The number of LFs comprising the combination in each study ranged from four to nine and included: smoking, alcohol, PA, sedentary time, television (TV) viewing time, various individual dietary factors, a dietary index, and sleep duration ( Table 1). Two studies included BMI in main analyses and one study included BMI in a sensitivity analysis ^43, 45, 46^. Alcohol and PA were included in all studies and dietary factors were missing from only one study ^45^. Smoking was included in five studies but excluded from relevant analyses in the remaining study ^44^. All LF data was collected *via* baseline questionnaire or interview.

### Definition or classification of unhealthy for individual lifestyle factors

In each study individual LFs were dichotomised as healthy/unhealthy with one point per factor summed to create an unweighted score. Two studies also created weighted scores using the strength of association between individual LFs and outcomes ^42, 46^. However, only one of these examined the effect of SES on a weighted score for which results were extracted here ^46^. Three studies summed healthy LFs to create ‘healthy’ scores ^42, 43, 46^, while the remaining three studies created ‘unhealthy’ scores (results were harmonised to show increasing risk with increasingly unhealthy lifestyle) ^19, 44, 45^.

The definition of unhealthy for each individual LF included in the LF combinations varied ( Table 1). Unhealthy smoking status was defined as current smoking ^19, 43^, current/any former smoking ^42^, current/quitting <20 years ago ^45^, and smoking more than 100 cigarettes in a lifetime ^46^. Unhealthy alcohol intake was defined as: >1 drink/day for women or >2 drinks/day for men ^42, 44– 46^, >5 drinks/day monthly ^45^, >46 g alcohol/day ^43^, and ‘daily/almost daily intake’, respectively ^19^. Unhealthy PA levels were defined as <150 minutes/week moderate or <75 minutes/week vigorous PA in four studies ^19, 42, 44, 45^, as strengthening activities on <2 days/week ^45^, as not achieving either ≥0.5 hours/day walking or ≥5 hours/week walking/playing sports ^43^, and as having leisure time PA levels in the lower two thirds of study participants ^46^. Unhealthy sedentary time, considered in two studies, was defined as the three quartiles with longest sedentary time ( *i.e.*, >5.75 and >6.5 hours/day), respectively ^42, 44^. Unhealthy TV viewing time, examined in one study, was defined as ≥4 hours/day ^19^. Unhealthy sleep duration, examined in two studies, was classified as <5.5/>7.4 hours/day ^43^ and <7/>9 hours/day ^19^, respectively.

Dietary factors examined varied considerably. Three studies of two USA-based cohorts used a national dietary index (comprising fruit, vegetables, grains, proteins, fatty acids, sodium, and calories from fats, alcohol, and added sugars), defining unhealthy as either belonging to the three lowest quartiles ^42, 44^ or two lowest quintiles ^46^. The Japanese cohort study included three dietary components, defining unhealthy as: fruit <once/day; fish <once/day; and milk <almost daily ^43^. One of the studies examining the UK-based UKB included four components, classifying unhealthy as: fruit and vegetables <400 g/day; oily fish <1 portion/week; red-meat >3 portion/week; and processed-meat >1 portion/week ^19^. Whereas the other study of UKB classified unhealthy as meeting at least five of 10 recommendations ^46^.

Justification for the classification of ‘unhealthy’ varied. One study cited WHO guidelines for the classification of unhealthy smoking ^42^. Four studies of USA-cohorts used US national guidelines to define unhealthy alcohol intake and diet ^42, 44– 46^. And of those, two also used US guidelines to define PA and sedentary time ^42, 44^. One study adapted a previous lifestyle score ^49^, using UK guidelines or standards from the original score ^19^. One study did not report the basis for their definitions of unhealthy for eight LFs including a BMI outwith 21–25 ^43^. The other study that examined BMI in their main analyses based the definition of unhealthy (<18.5 or ≥35) on prior analysis of the data ^45^. Unhealthy BMI (outwith 18.5–24.9) was based on previous research in the third study that included BMI in a sensitivity analysis ^46^.

Most studies had approximately normal distributions of the total number of unhealthy LFs among participants (S6 Table, which can be found as *Extended data*) ^37^. One study of UKB, with nine LFs, had relatively few participants with six to nine unhealthy LFs ^19^. The other study of UKB, with four LFs, had more participants with unhealthy LFs ^46^. The proportion of study participants with specific unhealthy LFs also varied. For example, the proportion of study participants with unhealthy smoking status ranged from 9.6% to 64%; some of this discrepancy is likely due to differences in the definition of unhealthy ( *i.e.*, current *versus* current/former smoking).

### Socioeconomic status

SES measures varied by study ( Table 1). For main analyses, two studies used area-based deprivation indices: Neighborhood deprivation index (NDI) and Townsend deprivation index (TDI) ^19, 44^. Data for both indices were obtained *via* national censuses from or near baseline. NDI comprises five ‘domains’: education, employment, housing, occupation, and poverty ^44^. Whereas TDI comprises data on car ownership, household overcrowding, owner occupation, and unemployment ^19^. Two studies used self-reported individual-level measures of income at recruitment ^42, 45^ and one of these operationalised income as a ratio of family income to the USA federal poverty level ^45^. One study used age at last formal education obtained *via* baseline self-report for the main analyses ^43^. Finally, one study of two cohorts used latent class analysis to generate an overall SES variable from four SES measures (income, occupation, education, and health insurance) in analysis of one cohort and three SES measures (income, education, and employment status) in analysis of the second cohort ^46^. In sensitivity analyses, two studies examined alternative SES measures ^19^. One study swapped area-based TDI for annual household income and, separately, individual-level educational attainment ^19^. The second study performed multiple sensitivity analyses of alternative SES measures by replacing a latent class SES variable with 1) each SES measure (income, occupation, education, health insurance, and employment status) used to generate the latent class; 2) an SES score based on each single SES measure; 3) and TDI ^46^.

### Categories for analysis

Categorisation of the two main exposures (combination of LFs and SES) used in analyses varied ( Table 1). Categories for combinations of LFs ranged from three to five and were not always related to the number of LFs included and often influenced by the number of participants with unhealthy LFs. For example, one study examined nine LFs and split participants into three categories: ‘healthy’ (score 0–2), ‘moderately healthy’ (score 3–5), and ‘unhealthy’ (score 6–9) ^19^; whereas another study included eight LFs and split participants into five categories ^43^.

For SES measures, the following categories were used: income dichotomised as </≥ $15,000 US dollars per annum ^42^; age at last formal education dichotomised as </≥ 16 years ^43^; quartiles of NDI ^44^; quintiles of TDI ^44^; ratio of family income to federal poverty level dichotomised as < or ≥200% of federal poverty level ^45^; three latent classes of low, medium and high SES ^46^.

### Analysis procedures

Each study conducted descriptive analyses, examining independent associations between combinations of LFs and outcomes and between SES and outcomes. All studies used Cox-proportional hazard models in their main analyses to estimate HRs and 95%CIs for outcomes for each LF combination category, stratified by SES ( Table 1). Three studies additionally stratified these analyses; one by ethnicity and sex together (African American/White and female/male) ^42^, three by sex alone ^43, 44, 46^, one by ethnicity (White/Non-white) ^46^, and one by age (≥60/<60 years) ^46^. One study that stratified by sex alone, also performed a separate analysis on the total population (not stratified by sex) ^43^. Two studies did not additionally stratify by sociodemographics ^19, 45^. The number of confounder variables chosen by studies ranged from five to 14 ( Table 2). All studies adjusted for either age, age plus age squared, or used age as the time-varying covariate.

**Table 2.** Methods/results for influence of SES on association between combinations of unhealthy LFs and outcomes.

Study	Methods	Covariates (n)	Interaction tests between combinations of unhealthy LFs and SES	Main interaction results (P _interaction or RERI_)	Result summary
**Andersen 2016 ^42^**	1) Cox-proportional hazard models for all-cause mortality for combination of unhealthy LF categories 2) Models stratified by low/high income in sub-group analysis	Enrolment source, education, marital status, neighbourhood deprivation, and BMI (5)	Likelihood ratio tests, comparing main effects models with and without cross-product terms	All-cause mortality: 0.002 (African American men); 0.89 (African American women); 0.04 (White men); 0.49 (White women)	Significant multiplicative interaction for African American and White men only: highest HRs for combination of unhealthy LFs and high income Only stratified (sex, ethnicity) results available
**Eguchi 2017 ^43^**	1) Cox-proportional hazard models for outcomes for combinations of unhealthy LF categories, stratified by low/high education (analyses for total population and separate analyses further stratified by sex) 2) Cox-proportional hazard models for combinations of unhealthy LF categories and education level using single reference group (all-cause and CVD mortality only) 3) Kaplan-Meier survival curves for combinations of unhealthy LF categories, stratified by low/high education (all-cause and CVD mortality only) 4) Sensitivity analysis examining two modified LF combinations	Age, sex, history of hypertension, history of diabetes, perceived mental stress and regular employment (6)	Cross-product of dichotomous education level and healthy lifestyle score (continuous) in models for total CVD and all-cause mortality outcomes only	All-cause mortality: 0.11 CVD mortality: 0.23 (both for total population only)	1) No evidence of multiplicative interaction, with similar HRs for combinations of unhealthy LFs and both high and low SES 2) Single reference group analysis provides evidence for additive interaction for all-cause and CVD mortality: higher HRs in least healthy combination of LFs and lowest education groups 3) Survival curves suggest additive interaction: steeper curve (highest mortality) for combination of unhealthy LFs in low education group 4) Sensitivity analysis: i) extended definition of healthy sleep and ii) dichotomous diet score (five components) in addition to extended sleep definition - consistent with findings from main analysis
**Andersen 2018 ^44^**	1) Cox-proportional hazard models for all-cause mortality for combinations of unhealthy LF categories, stratified by NDI quartiles 2) Cox-proportional hazard models for all-cause mortality for combinations of unhealthy LF categories, stratified by NDI quartiles using single reference (also stratified by sex)	Enrolment source, ethnicity, education, income, marital status, and insurance status (6)	Likelihood ratio tests, comparing main effects models with and without cross-product terms	All-cause mortality: 0.28 (men); 0.99 (women)	1) No evidence of multiplicative interaction with similar HRs for combinations of unhealthy LFs in both high and low SES 2) Single reference group analysis provides evidence for additive interaction in men and women for all-cause mortality: highest HRs in the least healthy combination of LFs and lowest SES (highest NDI) group
**Foster 2018 ^19^**	1) Cox-proportional hazard models for outcomes for combinations of unhealthy LF categories, stratified by SES quintiles (TDI, income, and education examined separately) 2) Cox-proportional hazard models for joint associations of combinations of unhealthy LF categories and SES measures (single reference group)	Age, sex, ethnicity, month of assessment, hypertension, systolic blood pressure, medication for hypercholesterolaemia or hypertension, and BMI (8)	1) Interaction term between combinations of unhealthy LFs and SES variables in models 2) Interaction sensitivity analyses (deprivation index): a) additional models with interaction term and i) dichotomous and ii) continuous combination of unhealthy LF variable b) Estimation of three measures of ‘biological interaction’: RERI, AP, and synergy index	Deprivation index All-cause and CVD- mortality: <0.0001 CVD incidence: 0.11 Income All-cause mortality: 0.001 CVD mortality: <0.0001 CVD incidence: 0.009 Education All-cause mortality: 0.002 CVD mortality: 0.047 CVD incidence: 0.051 (all for total population only)	1) Significant multiplicative interaction between combination of unhealthy LFs and deprivation/education for all-cause and CVD mortality but not for CVD incidence. Significant multiplicative interaction between combinations of unhealthy LFs and income for all outcomes 2) Single reference analysis showed highest HRs for all-cause and CVD mortality in the least healthy combination of LFs and lowest SES groups 3) Interaction sensitivity results consistent with main findings with significant interaction across three measures of additive interaction
**Choi 2022 ^45^**	1) Cox-proportional hazard models for all-cause mortality for number of unhealthy LFs, stratified by high/low income group	Age, age squared, sex, education, race/ethnicity, acculturation, income assistance, health insurance, and marital status (9)	Unclear, but likely an interaction term between combinations of unhealthy LFs and income in models	Primary outcomes All-cause mortality: <0.05	Significant multiplicative interaction between combinations of unhealthy LFs and income for all-cause mortality. Mortality risk associated with each additional unhealthy LF was higher in higher income group.
**Zhang 2021 ^46^**	1) Cox-proportional hazard models for outcomes for combinations of unhealthy LF categories, stratified by SES category 2) Cox-proportional hazard models for outcomes joint associations of combinations of unhealthy LF categories and SES measures (single reference group) 3) Sensitivity analyses for models stratified by SES category by examining subgroups: male/female, white/non-white ethnicity, age </≥60 years 4) Sensitivity analyses of joint associations substituting individual-level latent class SES for: a) Each SES component used to generate latent class, separately b) Townsend index (area-level) with adjustment for latent class SES (UKB only) and *vice versa*	Age, sex, marital status (NHANES only), assessment centre (UKB only), self-reported race/ethnicity, acculturation score, BMI, hypertension, diabetes, CVD, cancer, lung disease (UKB only). (10-14)	1) Interaction term between combinations of unhealthy LFs and SES variables in models 2) Estimation of RERI	All-cause mortality: 0.85; RERI =0 (NHANES), <0.001; RERI >0 (UKB) CVD mortality: 0.002; RERI >0 (UKB) CVD incidence: 0.016; RERI >0 (UKB) Secondary outcomes Heart disease mortality: 0.29; RERI =0 (NHANES) Coronary heart disease mortality: 0.008; RERI >0 (UKB) Stroke mortality: 0.002; RERI >0 (UKB) Myocardial infarction incidence: 0.050; RERI >0 (UKB) Stroke incidence: 0.032; RERI >0 (UKB)	1) NHANES: no significant multiplicative (product term for interaction) or additive interaction (RERI) between combination of unhealthy LFs and SES for all-cause or heart disease mortality UKB: both significant multiplicative and additive interactions between combination of unhealthy LFs and SES for all-cause mortality, CVD mortality, CVD incidence, coronary heart disease mortality, and stroke mortality but not for myocardial infarction incidence or stroke incidence 2) Results for product term for interaction and RERI similar across sensitivity analyses (individual-level SES, individual/area-level SES mutual adjustment) 3) In both cohorts, joint association analysis showed highest HRs in the least healthy combination of LFs and lowest SES groups for all outcomes and across all sensitivity analyses 4) Subgroup analyses showed significant multiplicative and additive interactions between combination of unhealthy LFs and SES for most subgroups (sex/ethnicity/age) and primary outcomes in UKB but not in NHANES 5) Subgroup analyses of the joint associations of combination of unhealthy LFs and SES showed higher HRs in men *vs.* women and in younger *vs.* older adults for all-cause mortality in both cohorts, and in younger *vs.* older adults for CVD mortality in UKB

LFs, lifestyle factors; SES, socioeconomic status; P _interaction_, p-value for interaction between combinations of unhealthy LFs and SES; RERI, relative excess risk due to interaction; HR, hazard ratio; NDI, Neighborhood deprivation index; TDI, Townsend deprivation index; BMI, body mass index; CVD, cardiovascular disease; ‘Biological interaction’, the degree of interaction between risk factors in terms of deviation from additivity in adverse health outcome rates ^50^; AP, attributable proportion; UKB, UK Biobank; NHANES, US National Health and Nutrition Examination Survey.

Studies varied in their additional analyses to investigate the influence of SES and included: single reference group analyses to investigate the joint associations of combinations of unhealthy LFs, SES, and outcomes ^19, 43, 44, 46^; Kaplan-Meier survival curves for combinations of unhealthy LFs stratified by SES ^43^; tests for multiplicative interactions between combinations of unhealthy LFs and SES ^19, 42– 46^; and tests for additive interactions ( Table 2) ^19, 46^.

### Study quality

Results for study quality as measured by the adapted NOS ranged from 5–9 (max. 9; S7 Table, which can be found as *Extended data*) ^37^. Only two studies examined more than one SES measure ^19, 46^ and only three studies attempted to reduce the chance of reverse causality by demonstrating participants were free from disease at the start of the study ^19, 43, 46^.

### The influence of socioeconomic status on lifestyle-associated health

**Figure 2.**
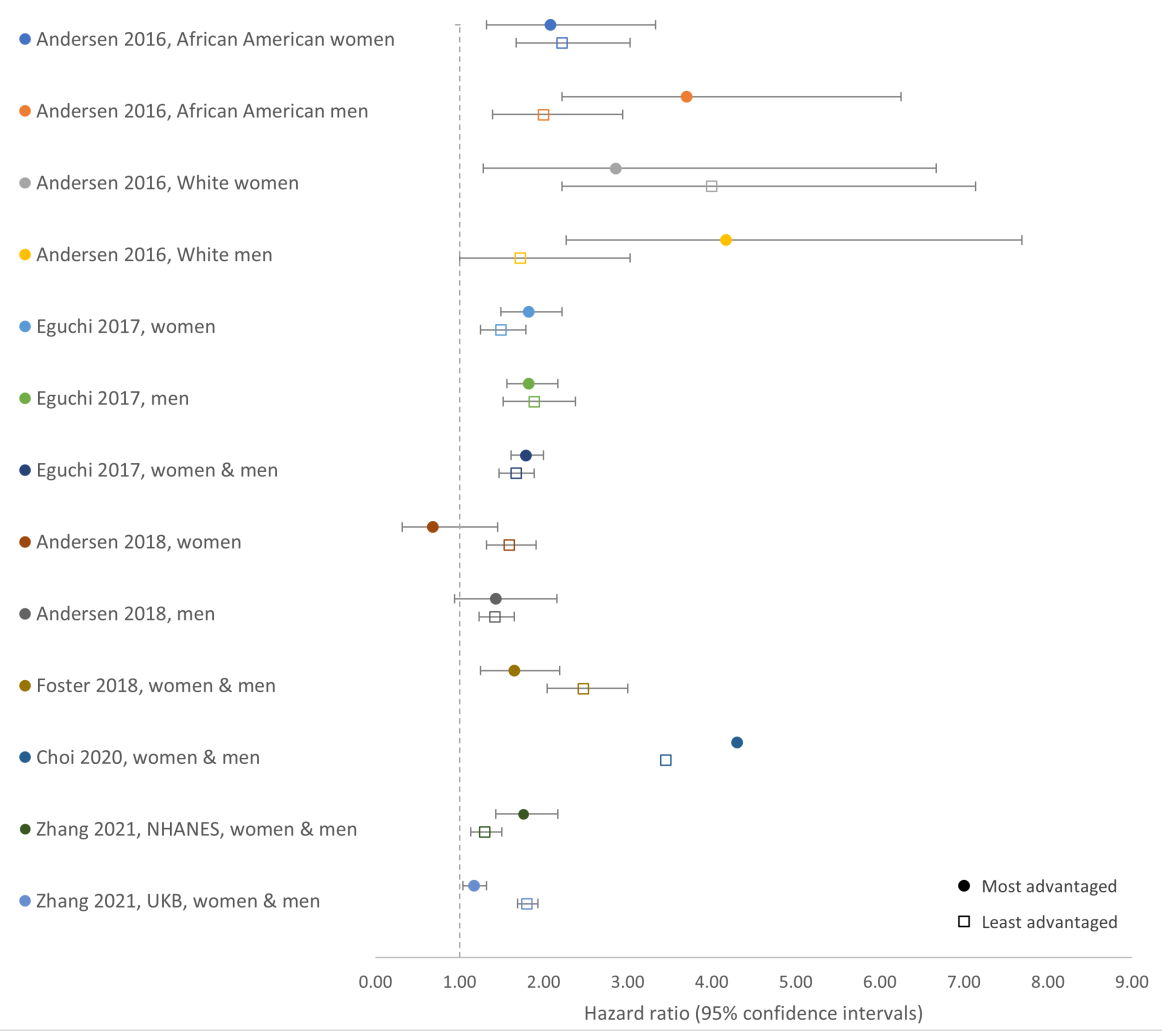
Hazard ratios for the association between combinations of unhealthy LFs and all-cause mortality in the most and least advantaged SES groups by study and population. Comparison of HRs from SES stratified analyses for the associations between combinations of unhealthy LFs and all-cause mortality in the most and least advantaged SES groups (main comparator). Combinations of healthy LFs in the same SES strata (most/least advantaged) are the reference group. LFs, lifestyle factors; SES, socioeconomic status; HR, hazard ratio; NHANES, US National Health and Nutrition Examination Survey; UKB, UK Biobank.

**Figure 3.**
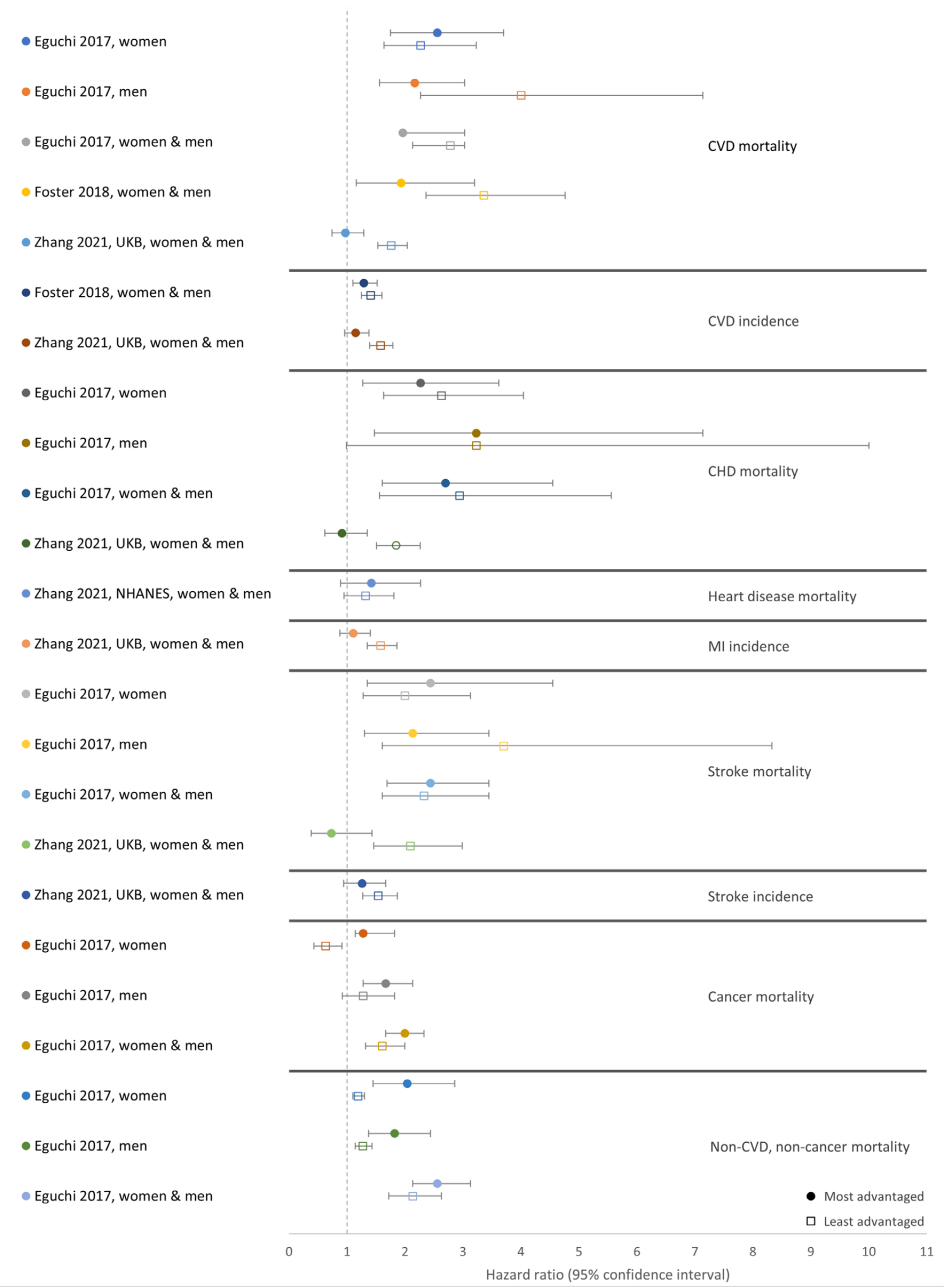
Hazard ratios for the association between combinations of unhealthy LFs and adverse health outcomes in the most and least advantaged SES groups by study and population. Comparison of HRs from SES stratified analyses for the associations between combinations of unhealthy LFs and adverse health outcomes in the most and least advantaged SES groups (main comparator). Combinations of healthy LFs in the same SES strata (most/least advantaged) are the reference group. LFs, lifestyle factors; SES, socioeconomic status; HR, hazard ratio; CVD, cardiovascular disease; CHD, coronary heart disease; MI, myocardial infarction; NHANES, US National Health and Nutrition Examination Survey; UKB, UK Biobank.

Using the main comparator as an assessment of the influence of SES on the association between combinations of unhealthy LFs and outcomes, results across studies were mixed and varied by outcome ( Figure 2 and Figure 3). A synthesis of results, including the main comparator, is structured by outcome below.

***All-cause mortality***. Estimates from 13 main analyses were available for the main comparator for all-cause mortality as some analyses were additionally stratified by sex or by both sex and ethnicity ( Figure 2). All studies observed that, compared with healthy LFs, combinations of unhealthy LFs were generally associated with higher all-cause mortality. However, the difference between the higher all-cause mortality associated with a combination of unhealthy *versus* that associated with healthy LFs was greater in the most advantaged SES group in seven analyses, but greater in the least advantaged group in the remaining six analyses ( Figure 2). However, there was considerable overlap of CIs from most and least advantaged SES groups and the difference between some estimates from most and least advantaged groups were similar. HRs (95%CIs) from the most advantaged groups ranged from 0.68 (0.32–1.45) to 4.17 (2.27–7.69); equivalent estimates from the least advantaged groups ranged from 1.30 (1.13–1.50) to 4.00 (2.22–7.14).

Two analyses were additionally stratified by sex alone and, for women, the difference in all-cause mortality associated with unhealthy *versus* healthy LFs was greater in the least advantaged group in one study ^44^ but greater in the most advantaged group in the other study ^43^ (and *vice versa* for men). The study that additionally stratified by both sex and ethnicity observed the difference in all-cause mortality associated with combinations of unhealthy *versus* healthy LFs was consistent for sex across two ethnic groups: greater in the least advantaged SES group for women of both African American and White ethnicity, but greater in the most advantaged SES group for men of both ethnicities ^42^. One study stratified by sex for sensitivity analysis and observed similar all-cause mortality associated with combinations of unhealthy *versus* healthy LFs for both sexes in the most *versus* least advantaged groups ^46^. However, the same study examined two cohorts and found that although the difference in all-cause mortality associated with combinations of unhealthy *versus* healthy LFs was small for men and women, it was greater in the most advantaged group in one cohort (NHANES) and in the least advantaged group in the other cohort (UKB) ^46^. Sensitivity analysis results from one study of two cohorts that additionally stratified by ethnicity alone (White/Non-White) were mixed ^46^. In the same study, sensitivity analysis stratified by age alone (≥60/<60 years old) suggested that all-cause mortality associated with combinations of unhealthy LFs was relatively higher for those <60 years old in the least advantaged groups in both cohorts ^46^. Five main analyses from four cohorts examined the total population (not further stratified by sociodemographic variables) and the difference in all-cause mortality associated with unhealthy *versus* healthy LFs was greater in the most advantaged SES group in three cohorts (JCCS, NHIS, NHANES) ^43, 45, 46^ but greater in the least advantaged group in another cohort (UKB) ^19, 46^. Similarly mixed results were found with the sensitivity analyses (S8 Table, which can be found as *Extended data*) ^37^.

Results of tests for multiplicative interactions were also mixed ( Table 2). A significant multiplicative interaction between the combination of LFs and SES was observed in four studies, but in opposing directions ^19, 42, 45, 46^. A significant multiplicative interaction was observed, with greater all-cause mortality associated with combinations of unhealthy LFs in the most advantaged group in the entire cohort of one study ^45^ but only in men in another study ^42^. Whereas a significant multiplicative interaction was seen in two studies of UKB, where the difference in all-cause mortality associated with unhealthy *versus* healthy LFs was greater in the least advantaged group ^19, 46^. The multiplicative interaction observed in UKB was observed consistently across a set of interaction sensitivity analyses ( Table 2) ^19, 46^. Two studies tested for and found significant additive interactions in the same cohort (UKB) ^19, 46^ but one of these studies did not observe significant additive interactions in similar analysis of a second cohort (NHANES) ^46^. Four studies of three cohorts examined combined associations of combinations of unhealthy LFs and less advantaged SES in eight analyses by comparing all groups to a single reference: the healthiest LF-most advantaged group ^19, 43, 44, 46^. In these analyses, HRs (95%CIs) for all-cause mortality for the least healthy-least advantaged group ranged from 1.43 (1.11–1.84) to 3.53 (3.01–4.14) (S9 Table, which can be found as *Extended data*) ^37^. The highest all-cause mortality was observed in the least healthy-least advantaged groups in seven of eight of these analyses, suggesting an additive interaction between unhealthy LFs and less advantaged SES. For sensitivity, two studies examined additional measures of SES separately in single reference group analyses and consistently observed the highest all-cause mortality in the least healthy-least advantaged groups irrespective of SES measure ^19, 46^. Further evidence for an additive interaction came from the steeper Kaplan-Meier curves for an unhealthy combination of LFs in the least advantaged *versus* most advantaged group in one study ^43^.

***CVD mortality***. Three studies examined CVD mortality in two cohorts. Compared with healthy LFs, combinations of unhealthy LFs were consistently associated with higher CVD mortality ^19, 43, 46^. In analyses stratified by SES alone, all three studies observed the difference in CVD mortality associated with unhealthy *versus* healthy LFs was greater in the least advantaged SES group: HRs (95%CIs) in the least advantaged groups were 2.78 (2.13–3.03) ^43^, 3.36 (2.36–4.76) ^19^, and 1.76 (1.53–2.04) ^46^, respectively. Equivalent estimates in the most advantaged groups were 1.96 (1.92–3.03), 1.93 (1.16–3.20), and 0.97 (0.74–1.29) ( Figure 3). One of these studies also stratified analyses by sex and found the difference in CVD mortality associated with unhealthy *versus* healthy LFs was greater in the most advantaged group for men but in the least advantaged group for women ^43^. However, the unhealthy *versus* healthy LFs CVD mortality for women was similar in the most and least advantaged groups. Similar results were found in sensitivity analyses (S8 Table, which can be found as *Extended data*) ^37^. For LF-SES interactions for CVD mortality, one study provided evidence of an additive interaction through both single reference group analyses and steeper Kaplan-Meier survival curves ^43^. In this study’s single reference group analysis, the highest CVD mortality was associated with those in the least healthy-least advantaged group (S10 Table, which can be found as *Extended data*) ^37, 43^. However, the same study found no significant multiplicative interaction for CVD mortality ( Table 2). By contrast, two other studies, both examining CVD mortality in UKB, reported a significant multiplicative interaction and in the single reference analysis, the least healthy-least advantaged group had markedly higher hazards than the least healthy-most advantaged group: 4.59 (3.33–6.32) *vs.* 2.01 (1.21–3.33) ^19^ and 2.65 (2.09–3.38) and 1.06 (0.80–1.39) ^46^, respectively (S10 Table, which can be found as *Extended data*) ^37^. Both studies observed significant multiplicative interactions for CVD mortality consistently irrespective of SES measure and across interaction sensitivity analyses ^19, 46^.

***Other outcomes***. Estimates for CVD incidence were provided by two studies of UKB and, in SES stratified analyses, compared with healthy LFs, combinations of unhealthy LFs were associated with higher CVD incidence ^19, 46^. The difference in CVD incidence associated with combinations of unhealthy *versus* healthy LFs was greater in the least advantaged groups in both studies. In combined single reference analysis, HRs (95%CIs) for the least healthy-most advantaged *versus* least healthy-least advantaged groups were: 1.30 (1.10–1.53) *versus* 1.75 (1.55–1.97) ^19^ and 1.18 (0.99–1.41) *versus* 2.09 (1.78–2.46) ^46^, respectively (S10 Table, which can be found as *Extended data*) ^37^. Results from tests for SES-LF interactions for CVD incidence were mixed. Significant additive and multiplicative interactions were observed in one study (examining four LFs and latent class SES) ^46^ but not the other (examining nine LFs and area-based TDI) ^19^.

Two studies examined additional outcomes ^43, 46^. One of these performed SES-stratified analyses but did not report single reference group analyses or tests for interaction for these outcomes ^43^. In this study’s SES-stratified analyses, the difference in hazards associated with combinations of unhealthy *versus* healthy LFs for the total population was greater in the most advantaged group for mortality from stroke, cancer, and non-CVD-non-cancer causes but greater in the least advantaged group for CHD mortality. Equivalent estimates from analyses additionally stratified by sex were similar, although, in men, the difference in hazards for stroke mortality was greater in the least advantaged group. In SES-stratified analyses in the second study that examined additional outcomes in two cohorts, the difference in hazards associated with combinations of unhealthy *versus* healthy LFs for the total population was greater in the most advantaged group for mortality from ‘heart disease’ in NHANES but greater in the least advantaged group for coronary heart disease and stroke, cancer, and stroke and myocardial infarction incidence in UKB ^46^.

## Discussion

Our review shows that the influence of SES on the association between a combination of unhealthy LFs and adverse health outcomes is unclear. There are several reasons for this. Firstly, few studies investigate this problem; only six studies met our eligibility criteria. Secondly, studies that do investigate this problem are heterogenous, varying by: cohort characteristics; lifestyle, SES, and covariate variables; outcomes assessed; and methodology by which SES influence was examined. Thirdly, where broadly similar estimates were compared directly ( *i.e.*, *via* our main comparator), results were mixed: the difference in hazards associated with combinations of unhealthy *versus* healthy LFs was greater in the most advantaged SES group for some studies or cohorts and outcomes but in the least advantaged group for others. Fourthly, results for tests for multiplicative interactions between combinations of LFs and SES were conflicting. For example, for all-cause mortality, two studies found no evidence of multiplicative interaction ^43, 44^; two studies reported significant multiplicative interactions but observed a moderating influence of SES in opposing directions ^19, 42^; while a fifth study, of two cohorts, found significant multiplicative interactions in one cohort but not the other ^46^. Finally, the quality of included studies varied, with only one scoring the highest possible quality score, so available study estimates may be biased.

The heterogeneity of the LF and SES exposure variables examined by the included studies warrants further discussion. Risk estimates associated with combinations of different LFs are difficult to compare where combinations from different studies lack shared LFs ( *e.g.*, combination 1: smoking, alcohol, and physical inactivity *vs.* combination 2: sedentary time, unhealthy diet, and sleep duration). Further, each LF will have differential contributions to the level of risk associated with the overall combination ( *e.g.*, smoking is likely to drive the largest share of risk associated with CVD mortality) ^51^, thus making comparisons of estimates associated with unweighted combinations of different LFs hard to interpret. However, risk estimates associated with the healthiest and unhealthiest LF combinations where studies share similar LF components ( *e.g.*, combination 1: alcohol, unhealthy diet, and physical inactivity *vs.* combination 2: alcohol, unhealthy diet, physical inactivity, and smoking), are more comparable. Moreover, the aim of this review was to identify and appraise all studies that examined the effect of SES on the association between any LF combination and adverse health. Restricting the searches of this review to identify only those studies with the same or similar combinations of LFs would have yielded fewer results. The rationale to identify studies examining SES effects on the association between combinations of LFs and adverse health is based on the known clustering of LFs and the consequent higher associated risks ^4– 8, 52^. Future research could attempt to identify highest risk LF combinations, and whether the riskiest combinations vary by SES. This could provide new targets for intervention and inform policies attempting to address unhealthy LFs in the least advantaged sections of society ^53^.

The range of SES measures used across studies highlights the myriad ways in which SES can be measured and ranked ^11^. Although there is likely to be a high degree of correlation across SES measures, the impacts of different SES measures on the association between combinations of LFs and adverse health could be different ^54^. For example, an individual-level measure ( *e.g.*, age at last formal education) could have a weaker modifying effect on the association between combinations of LFs and adverse health than an area-based deprivation index if wider socioeconomic factors included or captured by the index (directly or indirectly) have a greater effect on the association. For instance, proximity and access to healthy food or green spaces for PA could be more strongly associated with area-based SES indices than with individual-level SES measures ^55^. Irrespective of SES heterogeneity, if an effect of SES was identified that was consistent across a broad range of SES measures this would strengthen the evidence for a general SES effect. Whereas if SES effects were consistently associated with one type of SES measurement ( *e.g.*, income) and not others ( *e.g.*, area-based indices) this could generate hypotheses and inform research that aims to explain underlying mechanisms of SES effects ^54^. The aim of this review was to identify all available evidence and therefore studies were not excluded on the basis of LF and SES exposure variables despite the expected difficulties in comparability.

Notwithstanding study heterogeneity and the lack of data, the studies’ assessments of the influence of SES on the association between a combination of unhealthy LFs and adverse health outcomes point broadly towards an additive influence of SES. Examining the combined effect of SES and combinations of unhealthy LFs by way of a single reference group (the healthiest LF-most advantaged group), four studies of five cohorts provide evidence for an additive interaction for multiple outcomes ^19, 43, 44, 46^. Two of these studies, both examining UKB, also observed significant results from formal tests for additive interactions as well as significant multiplicative interactions in same direction ^19, 46^. Together, this evidence does not strongly support a vulnerability hypothesis but it does provide some evidence against the so-called Blaxter hypothesis ^56^. The Blaxter hypothesis suggests that detrimental effects of unhealthy lifestyles are masked by other adverse factors also associated with less advantaged SES ( *e.g.*, insecure income, poor housing, more frequent adverse childhood experiences). If this hypothesis were correct, in analyses stratified by SES and in least advantaged SES groups, associations between combinations of LFs and adverse health would be similar whether the LFs were healthy or unhealthy ( *i.e.*, a combination of unhealthy LFs would have little influence on a population with an already high risk due to other factors). However, in all studies, compared to those with healthy LFs, there were higher hazards for adverse health outcomes in those with a combination of unhealthy LFs irrespective of SES level. One study observed a multiplicative interaction (in men only), where the difference in hazards associated with a combination of unhealthy *versus* healthy LFs was greater in the most advantaged SES group, which could support the Blaxter hypothesis ^42^. However, the authors did not report a single reference group analysis, which could help clarify the combined associations. Overall, the impression of an additive interaction between least advantaged SES and combinations of unhealthy LFs seen in four studies of five cohorts and a multiplicative interaction in the same direction in two studies suggests that the detrimental effects of combinations of unhealthy LFs are not masked by other harmful factors associated with less advantaged SES but are at least in addition to, and potentially synergistic with, those factors. This finding, if borne out in future research, would indicate that less advantaged SES populations have the highest absolute risks associated with combinations of unhealthy LFs and would, therefore, support a strategy of focussing lifestyle resources on less advantaged SES populations where need is greatest.

### Strengths and limitations

This review is strengthened by a rigorous pre-specified protocol ^35^; a comprehensive search strategy including database, reference, citation, and grey literature searches ^36^; and by reviewers working independently. Further, data synthesis follows SWiM guidelines and is fully transparent ^41^. However, this review is limited by the small number of studies included and by the high level of heterogeneity between studies, which precluded meta-analysis. Therefore, the conclusions drawn here about whether and how SES influences the association between combinations of unhealthy LFs and adverse health may be altered by future research. Importantly, differential vulnerability to combinations of unhealthy LFs could be due to differential exposure that is not captured *via* questionnaires. For example, excess alcohol in less advantaged SES populations may be more extreme than excess alcohol in more advantaged groups ^57^. Similarly, residual confounding, with unaccounted for differences between more and less advantaged populations, could also explain observed differential vulnerability. Our eligibility criteria may have been too restrictive resulting in few studies and retrospective studies may have yielded additional evidence. However, the level of evidence from retrospective design is lower. Future prospective studies, where data are updated during follow-up, could reduce potential misclassification bias by capturing participants’ lifestyle changes. While the adverse health outcomes included here account for the vast majority of mortality and NCD burden ^58^, others, such as dementia and renal disease, are growing in prevalence and have similar lifestyle risk factors ^59, 60^. The aim of this review was to identify and synthesise the evidence for SES modification of associations between LF combinations and adverse health outcomes, not to explain any identified effect modification. However, strong evidence for SES effect modification of such associations could prompt attempts to uncover underlying mechanisms, such as cumulative risks or accelerated biological ageing ^25– 27^.

## Conclusions

This is the first systematic review to examine if and how SES modifies associations between combinations of unhealthy LFs and adverse health outcomes. Prospective studies that examine this problem are few and heterogenous. The influence of SES on lifestyle-associated adverse health could be additive but remains unclear. New research using multiple datasets, a range of lifestyle and SES measures, and a comprehensive list of adverse health outcomes would improve understanding of SES influence on lifestyle risks and thereby inform lifestyle-related policy and interventions.

## Data Availability

All data underlying the results are available as part of the article and no additional source data are required. Figshare: 2022_12_08_SES_lifestyle_systematic_rv_SUPPORTING_INFORMATION.docx. https://doi.org/10.6084/m9.figshare.21701519 ^37^. Figshare: PRISMA checklists for the abstract and main manuscript of ‘The influence of socioeconomic status on the association between unhealthy lifestyle factors and adverse health outcomes: a systematic review’. https://doi.org/10.6084/m9.figshare.21770651 ^33^ and https://doi.org/10.6084/m9.figshare.21770657 ^34^. Data are available under the terms of the Creative Commons Attribution 4.0 International license (CC-BY 4.0).

## References

[1] GBD 2017 Risk Factor Collaborators: Global, regional, and national comparative risk assessment of 84 behavioural, environmental and occupational, and metabolic risks or clusters of risks for 195 countries and territories, 1990-2017: a systematic analysis for the Global Burden of Disease Study 2017. *Lancet.* 2018;392(10159):1923–94. 10.1016/S0140-6736(18)32225-6 30496105 PMC6227755

[2] Zhang YB Pan XF Chen J : Combined lifestyle factors, all-cause mortality and cardiovascular disease: a systematic review and meta-analysis of prospective cohort studies. *J Epidemiol Community Health.* 2021;75(1):92–99. 10.1136/jech-2020-214050 32892156

[3] Zhang YB Pan XF Chen J : Combined lifestyle factors, incident cancer, and cancer mortality: a systematic review and meta-analysis of prospective cohort studies. *Br J Cancer.* 2020;122(7):1085–93. 10.1038/s41416-020-0741-x 32037402 PMC7109112

[4] Meader N King K Moe-Byrne T : A systematic review on the clustering and co-occurrence of multiple risk behaviours. *Bmc Public Health.* 2016;16:657. 10.1186/s12889-016-3373-6 27473458 PMC4966774

[5] Noble N Paul C Turon H : Which modifiable health risk behaviours are related? A systematic review of the clustering of Smoking, Nutrition, Alcohol and Physical activity ('SNAP') health risk factors. *Prev Med.* 2015;81:16–41. 10.1016/j.ypmed.2015.07.003 26190368

[6] Hart CL Davey Smith G Gruer L : The combined effect of smoking tobacco and drinking alcohol on cause-specific mortality: a 30 year cohort study. *Bmc Public Health.* 2010;10:789. 10.1186/1471-2458-10-789 21184680 PMC3022858

[7] Ford ES Bergmann MM Boeing H : Healthy lifestyle behaviors and all-cause mortality among adults in the United States. *Prev Med.* 2012;55(1):23–7. 10.1016/j.ypmed.2012.04.016 22564893 PMC4688898

[8] Behrens G Fischer B Kohler S : Healthy lifestyle behaviors and decreased risk of mortality in a large prospective study of U.S. women and men. *Eur J Epidemiol.* 2013;28(5):361–72. 10.1007/s10654-013-9796-9 23532745

[9] McNamara CL Balaj M Thomson KH : The socioeconomic distribution of non-communicable diseases in Europe: findings from the European Social Survey (2014) special module on the social determinants of health. *Eur J Public Health.* 2017;27(suppl_1):22–6. 10.1093/eurpub/ckw222 28355638

[10] Inequalities in Health: Report of a Research Working Group ('The Black report'). Department of Health and Social Security, London (UK).1980.

[11] Oakes JM Rossi PH : The measurement of SES in health research: current practice and steps toward a new approach. *Soc Sci Med.* 2003;56(4):769–84. 10.1016/s0277-9536(02)00073-4 12560010

[12] Darin-Mattsson A Fors S Kareholt I : Different indicators of socioeconomic status and their relative importance as determinants of health in old age. *Int J Equity Health.* 2017;16(1):173. 10.1186/s12939-017-0670-3 28950875 PMC5615765

[13] Smith GD Hart C Hole D : Education and occupational social class: which is the more important indicator of mortality risk? *J Epidemiol Community Health.* 1998;52(3):153–60. 10.1136/jech.52.3.153 9616419 PMC1756692

[14] Stringhini S Carmeli C Jokela M : Socioeconomic status, non-communicable disease risk factors, and walking speed in older adults: multi-cohort population based study. *BMJ.* 2018;360:k1046. 10.1136/bmj.k1046 29572376 PMC5865179

[15] Marmot MG Shipley MJ Hemingway H : Biological and behavioural explanations of social inequalities in coronary heart disease: the Whitehall II study. *Diabetologia.* 2008;51(11):1980–8. 10.1007/s00125-008-1144-3 18777168 PMC2788759

[16] Laine JE Baltar VT Stringini S : Reducing socio-economic inequalities in all-cause mortality: a counterfactual mediation approach. *Int J Epidemiol.* 2019;49(2):497–510. 10.1093/ije/dyz248 31855265 PMC7266549

[17] Nordahl H Lange T Osler M : Education and cause-specific mortality: the mediating role of differential exposure and vulnerability to behavioral risk factors. *Epidemiology.* 2014;25(3):389–96. 10.1097/EDE.0000000000000080 24625538

[18] Bihan H Backholer K Peeters A : Socioeconomic Position and Premature Mortality in the AusDiab Cohort of Australian Adults. *Am J Public Health.* 2016;106(3):470–7. 10.2105/AJPH.2015.302984 26794164 PMC4815750

[19] Foster HME Celis-Morales CA Nicholl BI : The effect of socioeconomic deprivation on the association between an extended measurement of unhealthy lifestyle factors and health outcomes: a prospective analysis of the UK Biobank cohort. *Lancet Public Health.* 2018;3(12):e576–e85. 10.1016/S2468-2667(18)30200-7 30467019

[20] Pampel FC Rogers RG : Socioeconomic status, smoking, and health: A test of competing theories of cumulative advantage. *J Health Soc Behav.* 2004;45(3):306–21. 10.1177/002214650404500305 15595509

[21] Katikireddi SV Whitley E Lewsey J : Socioeconomic status as an effect modifier of alcohol consumption and harm: analysis of linked cohort data. *Lancet Public Health.* 2017;2(6):E267–E76. 10.1016/S2468-2667(17)30078-6 28626829 PMC5463030

[22] Birch S Jerrett M Eyles J : Heterogeneity in the determinants of health and illness: the example of socioeconomic status and smoking. *Soc Sci Med.* 2000;51(2):307–17. 10.1016/S0277-9536(99)00455-4 10832576

[23] Foster HME Ho FK Mair FS : The association between a lifestyle score, socioeconomic status, and COVID-19 outcomes within the UK Biobank cohort. *Bmc Infect Dis.* 2022;22(1):273. 10.1186/s12879-022-07132-9 35351028 PMC8964028

[24] Diderichsen F Hallqvist J Whitehead M : Differential vulnerability and susceptibility: how to make use of recent development in our understanding of mediation and interaction to tackle health inequalities. *Int J Epidemiol.* 2019;48(1):268–74. 10.1093/ije/dyy167 30085114

[25] Belsky DW Caspi A Cohen HJ : Impact of early personal-history characteristics on the Pace of Aging: implications for clinical trials of therapies to slow aging and extend healthspan. *Aging Cell.* 2017;16(4):644–51. 10.1111/acel.12591 28401731 PMC5506399

[26] Fiorito G Polidoro S Dugué PA : Social adversity and epigenetic aging: a multi-cohort study on socioeconomic differences in peripheral blood DNA methylation. *Sci Rep.* 2017;7(1):16266. 10.1038/s41598-017-16391-5 29176660 PMC5701128

[27] Pool U : Socioeconomic inequalities in lifestyle-related health outcomes. *Lancet Public Health.* 2019;4(2):e85. 10.1016/S2468-2667(19)30003-9 30738506

[28] Mackenbach JD Brage S Forouhi NG : Does the importance of dietary costs for fruit and vegetable intake vary by socioeconomic position? *Br J Nutr.* 2015;114(9):1464–70. 10.1017/S0007114515003025 26353803 PMC4657115

[29] Burgoine T Mackenbach JD Lakerveld J : Interplay of Socioeconomic Status and Supermarket Distance Is Associated with Excess Obesity Risk: A UK Cross-Sectional Study. *Int J Environ Res Public Health.* 2017;14(11):1290. 10.3390/ijerph14111290 29068365 PMC5707929

[30] Pepper GV Nettle D : The behavioural constellation of deprivation: Causes and consequences. *Behav Brain Sci.* 2017;40:e314. 10.1017/S0140525X1600234X 28073390

[31] Wardle J Steptoe A : Socioeconomic differences in attitudes and beliefs about healthy lifestyles. *J Epidemiol Community Health.* 2003;57(6):440–3. 10.1136/jech.57.6.440 12775791 PMC1732468

[32] Moher D Liberati A Tetzlaff J : Preferred reporting items for systematic reviews and meta-analyses: the PRISMA statement. *BMJ.* 2009;339:b2535. 10.1136/bmj.b2535 19622551 PMC2714657

[33] Foster H : 2022_ 12_ 22_PRISMA_ 2020_abstract_checklist.docx. Figshare. [Dataset].2022. 10.6084/m9.figshare.21770651.v1

[34] Foster H : PRISMA 2020 Checklist. Figshare[Dataset].2022. 10.6084/m9.figshare.21770657.v1

[35] Foster H Polz P Mair F : Understanding the impact of socioeconomic status on the association between combined lifestyle factors and adverse health outcomes: a systematic review.PROSPERO 2020 CRD42020172588, PROSPERO - International prospective register of systematic reviews;2020. Reference Source

[36] Foster H Polz P Mair FS : Understanding the influence of socioeconomic status on the association between combinations of lifestyle factors and adverse health outcomes: a systematic review protocol. *BMJ Open.* 2021;11(5):e042212. 10.1136/bmjopen-2020-042212 34045211 PMC8162079

[37] Foster H : 2022_ 12_ 08_SES_lifestyle_systematic_rv_SUPPORTING_INFORMATION.docx. Figshare. [Dataset].2022. 10.6084/m9.figshare.21701519.v1

[38] Zhang Y Pan XF Chen J : Combined lifestyle factors and risk of incident type 2 diabetes and prognosis among individuals with type 2 diabetes: a systematic review and meta-analysis of prospective cohort studies. *Diabetologia.* 2020;63(1):21–33. 10.1007/s00125-019-04985-9 31482198

[39] McKenzie J Brennan SE Ryan RE : Chapter 3: Defining the criteria for including studies and how they will be grouped for the synthesis.In: *Cochrane Handbook for Systematic Reviews of Interventions version 6.0.* 2019. 10.1002/9781119536604.ch3

[40] Wells G Shea B O'Connell D : The Newcastle-Ottawa Scale (NOS) for assessing the quality of nonrandomised studies in meta-analyses.Ottawa Hospital Research Institute.2019. Reference Source

[41] Campbell M McKenzie JE Sowden A : Synthesis without meta-analysis (SWiM) in systematic reviews: reporting guideline. *BMJ.* 2020;368:l6890. 10.1136/bmj.l6890 31948937 PMC7190266

[42] Andersen SW Zheng W Sonderman J : Combined Impact of Health Behaviors on Mortality in Low-Income Americans. *Am J Prev Med.* 2016;51(3):344–55. 10.1016/j.amepre.2016.03.018 27180031 PMC4992598

[43] Eguchi E Iso H Honjo K : No modifying effect of education level on the association between lifestyle behaviors and cardiovascular mortality: the Japan Collaborative Cohort Study. *Sci Rep.* 2017;7:39820. 10.1038/srep39820 28057921 PMC5216353

[44] Andersen SW Blot WJ Shu XO : Associations Between Neighborhood Environment, Health Behaviors, and Mortality. *Am J Prev Med.* 2018;54(1):87–95. 10.1016/j.amepre.2017.09.002 29254556 PMC5739075

[45] Choi SH Stommel M Ling J : The Impact of Smoking and Multiple Health Behaviors on All-Cause Mortality. *Behav Med.* 2022;48(1):10–7. 10.1080/08964289.2020.1796570 32701418

[46] Zhang YB Chen C Pan XF : Associations of healthy lifestyle and socioeconomic status with mortality and incident cardiovascular disease: two prospective cohort studies. *BMJ.* 2021;373:n604. 10.1136/bmj.n604 33853828 PMC8044922

[47] About the National Health Interview Survey.(accessed 31/03/2022. Reference Source

[48] About the National Health and Nutrition Examination Survey.(accessed 31/03/2022. Reference Source

[49] Ding D Rogers K van der Ploeg H : Traditional and Emerging Lifestyle Risk Behaviors and All-Cause Mortality in Middle-Aged and Older Adults: Evidence from a Large Population-Based Australian Cohort. *PLoS Med.* 2015;12(12):e1001917. 10.1371/journal.pmed.1001917 26645683 PMC4672919

[50] Andersson T Alfredsson L Källberg H : Calculating measures of biological interaction. *Eur J Epidemiol.* 2005;20(7):575–9. 10.1007/s10654-005-7835-x 16119429

[51] Yusuf S Joseph P Rangarajan S : Modifiable risk factors, cardiovascular disease, and mortality in 155 722 individuals from 21 high-income, middle-income, and low-income countries (PURE): a prospective cohort study. *Lancet.* 2020;395(10226):795–808. 10.1016/S0140-6736(19)32008-2 31492503 PMC8006904

[52] Buck D Frosini F : Clustering of unhealthy behaviours over time. Implications for policy and practice.The Kings Fund,2012. Reference Source

[53] Evans H Buck D : Tackling multiple unhealthy risk factors. Emerging lessons from practice.The Kings Fund,2018. Reference Source

[54] Galobardes B Lynch J Smith GD : Measuring socioeconomic position in health research. *Br Med Bull.* 2007;81–82:21–37. 10.1093/bmb/ldm001 17284541

[55] Kabisch N : The Influence of Socio-economic and Socio-demographic Factors in the Association Between Urban Green Space and Health.In: Marselle M. SJ, Korn H., Irvine K., Bonn A., ed. *Biodiversity and Health in the Face of Climate Change.* Springer, Cham.;2019;91–119. 10.1007/978-3-030-02318-8_5

[56] Blaxter M : Health and lifestyles.London; New York: Routledge;1990. 10.4324/9780203393000

[57] Lewer D Meier P Beard E : Unravelling the alcohol harm paradox: a population-based study of social gradients across very heavy drinking thresholds. *BMC Public Health.* 2016;16:599. 10.1186/s12889-016-3265-9 27430342 PMC4950253

[58] GBD 2019 Diseases and Injuries Collaborators: Global burden of 369 diseases and injuries in 204 countries and territories, 1990-2019: a systematic analysis for the Global Burden of Disease Study 2019. *Lancet.* 2020;396(10258):1204–22. 10.1016/S0140-6736(20)30925-9 33069326 PMC7567026

[59] Peters R Booth A Rockwood K : Combining modifiable risk factors and risk of dementia: a systematic review and meta-analysis. *BMJ Open.* 2019;9(1):e022846. 10.1136/bmjopen-2018-022846 30782689 PMC6352772

[60] Wakasugi M Kazama JJ Yamamoto S : A combination of healthy lifestyle factors is associated with a decreased incidence of chronic kidney disease: a population-based cohort study. *Hypertens Res.* 2013;36(4):328–33. 10.1038/hr.2012.186 23171953

