# Supplementary material for: The influence of socioeconomic status on the association between unhealthy lifestyle factors and adverse health outcomes: a systematic review
Source: Wellcome Open Res. 2023 Dec 8;8:55. Originally published 2023 Feb 3. [Version 2] doi: 10.12688/wellcomeopenres.18708.2 (PMC10964004; doi:10.12688/wellcomeopenres.18708.2)
Supplement: Peer review history

# Reviewer report: Reviewer response for version 2

**DOI:** 10.21956/wellcomeopenres.22269.r75849

**Published:** 2024-03-25

**Authors:** Ming-Jie Duan

**Affiliations:** 1 Human Nutrition & Health, Wageningen University & Research, Wageningen, Gelderland, The Netherlands; 2 Department of Internal Medicine, University Medical Center Groningen, Groningen, The Netherlands

Thanks for inviting me to review this manuscript. Foster et al performed a systematic review to examine whether socioeconomic status modifies the associations between lifestyle factors and health outcomes. The manuscript is well-written, and the topic is inspiring, addressing an important question.

 Limitations of this study include the limited number of available studies and the strict inclusion criteria. The authors have addressed these limitations; however, I would recommend that they further fine-tune the discussion here. Specifically, what is the potential influence on the study results as only studies examining combinations of lifestyle factors are included? Indeed, one can argue that there are synergistic/interactive effects between lifestyle factors. However, such effects cannot be simply accounted for by examining an unweighted lifestyle score, which was used in most of the studies. In fact, studies focusing on only a single lifestyle factor (such as diet quality) also show that SES modifies the associations between the lifestyle factor and health outcomes, controlling for other lifestyle factors. Additionally, alcohol consumption may be higher in highly educated groups than in less educated groups and might be protective for cardiovascular health. I was wondering what the authors’ opinions are on this.

 Furthermore, this study has important public health implications, which may be discussed more extensively in the manuscript. The current discussion focuses mostly on methodological details and explanations of the potential mechanisms of the findings (which is good), while policy implications are quite limited.

 A final small comment is on Figure 3. I understand the authors' intention to show the difference in the gradient of the associations across SES groups. Nevertheless, this figure, although concise, may lose important information, such as the additive (or in some studies multiplicative) effects of SES and unhealthy lifestyle on health outcomes, compared to the group that is most advantaged with the healthiest lifestyle, as well as whether the associations between lifestyle and health outcomes follow a dose-response manner.

Are the rationale for, and objectives of, the Systematic Review clearly stated?

Yes

Is the statistical analysis and its interpretation appropriate?

Yes

Are sufficient details of the methods and analysis provided to allow replication by others?

Yes

Are the conclusions drawn adequately supported by the results presented in the review?

Yes

Reviewer Expertise:

Epidemiology, public health, nutrition.

I confirm that I have read this submission and believe that I have an appropriate level of expertise to confirm that it is of an acceptable scientific standard.

# Reviewer report: Reviewer response for version 2

**DOI:** 10.21956/wellcomeopenres.22269.r71126

**Published:** 2023-12-28

**Authors:** Nuno Mendonça

**Affiliations:** 1 CHRC, Universidade Nova de Lisboa, Lisbon, Portugal; 2 NOVA Medical School, Universidade Nova de Lisboa (NMS/UNL), Universidade Nova de Lisboa (NMS/UNL), Lisboa, Portugal

The rebuttal addressed most of my concerns and importantly told me that the authors thought about those issues. I accept the manuscript without any further changes.

Are the rationale for, and objectives of, the Systematic Review clearly stated?

Yes

Is the statistical analysis and its interpretation appropriate?

Yes

Are sufficient details of the methods and analysis provided to allow replication by others?

Yes

Are the conclusions drawn adequately supported by the results presented in the review?

Yes

Reviewer Expertise:

Nutrition, epidemiology, ageing

I confirm that I have read this submission and believe that I have an appropriate level of expertise to confirm that it is of an acceptable scientific standard.

# Reviewer report: Reviewer response for version 1

**DOI:** 10.21956/wellcomeopenres.20745.r58260

**Published:** 2023-07-14

**Authors:** Nuno Mendonça

**Affiliations:** 1 CHRC, Universidade Nova de Lisboa, Lisbon, Portugal; 2 NOVA Medical School, Universidade Nova de Lisboa (NMS/UNL), Universidade Nova de Lisboa (NMS/UNL), Lisboa, Portugal

I thank the authors for the big amount of work that went into searching, reviewing, analysing and drafting “The influence of socioeconomic status on the association between unhealthy lifestyle factors and adverse health outcomes: a systematic review”. The paper is well-written and there is a careful presentation of the results. However, I have several concerns/comments that I will list, not by order of importance, but by order of appearance.

  

1. The title mentions “adverse health outcomes” but I think that a better description of what those outcomes are would be more informative. It could read “The influence of socioeconomic status on the association between unhealthy lifestyle factors and cardiovascular disease, cancer and mortality: a systematic review”.
2. The search was conducted 1.5 years ago and although I am aware of the work involved in running it again I believe that, unless there is a very good reason not to, there should at the very least some mention in the discussion of the number of papers published that met your inclusion criteria in the past 1.5 years.
3. There are other databases that could have been searched as well. I believe the authors should justify why there was a decision not to search them, even if that decision was time constraints and believing that all the studies would be included in PubMed/EMBASE/CINAHL.
4. Abstract: It would be good to describe briefly which LFs were used or something from this phrase “i. combination of ≥3 LFs: studies that also included metabolic/intermediate factors ( *e.g.*, blood pressure/body mass index (BMI)) as part of their combination of LFs were included so long as the combination also included ≥3 ‘behavioural’ LFs ( *e.g.*, smoking/PA/diet).”
5. The protocol mentions that all-cause mortality was the main outcome but I didn’t seem to find any mention of it in this paper.
6. Introduction: The authors mention different possible explanations but I missed the authors hypothesis of how SES plays moderates the association between lifestyle and health outcomes, or mention which (or a blend of both) of the existing explanations is favoured by the authors.
7. Figure 2. Most of the HRs are between 1 and 3 but the ticks on the x axis are only every 1-point which doesn’t let me, from the figure alone, see the estimates but only the direction and general magnitude. I suggest adding more ticks and having 1 decimal point instead of 2 (also remember its a log scale). And/or (and ideally) having the HRs and corresponding 95%CI on the plot for every row/analysis. I see that Choi 2020 did not provide confidence intervals or any measure of variability but that should be mentioned in the figure notes.
8. Supplementary tables should be mentioned along the text when needed .e.g. there is no mention on Table S8 when reporting on the results of the association between combinations of unhealthy LFs and all-cause mortality (figure 2). Also supplementary material are not annexes and table/figures/text should only be included in the paper when these are mentioned.
9. Check if abbreviations are defined at their first appearance, .e.g NHANES in the abstract.
10. Discussion: There should be some mention about the possible selection bias in the recruitment of these studies, i.e. it is notoriously harder to recruit people from lower SES status.
11. Discussion: The authors mention how different combinations of LFs were used across studies but these were also measured differently. Please add that besides the difficulty of comparing different combinations of LFs, these were also often times measured in a very different way.
12. Discussion: Not using individual lifestyle such as smoking or alcohol and instead using lifestyle, combination, etc may have meant that some papers were not picked up. If this was the case or if the authors think this was unlikely, it should be mentioned
13. Discussion: “However, the level of evidence from retrospective design is lower” should be taken with caution because it oversimplifies the quality of evidence based on simply the study design. Adding something like “observational retrospective design may, in general, have more biases than a well-designed prospective observational study” would be better.

Are the rationale for, and objectives of, the Systematic Review clearly stated?

Yes

Is the statistical analysis and its interpretation appropriate?

Yes

Are sufficient details of the methods and analysis provided to allow replication by others?

Yes

Are the conclusions drawn adequately supported by the results presented in the review?

Yes

Reviewer Expertise:

Nutrition, epidemiology, ageing

I confirm that I have read this submission and believe that I have an appropriate level of expertise to confirm that it is of an acceptable scientific standard, however I have significant reservations, as outlined above.

# Response

**Published:** 2023-11-28

**Authors:** Hamish Foster

**Affiliations:** University of Glasgow, UK

**2 ^nd^ Reviewer - Nuno Mendonça, CHRC, NOVA Medical School, Universidade Nova de Lisboa, Lisboa, Portugal **   I thank the authors for the big amount of work that went into searching, reviewing, analysing and drafting “The influence of socioeconomic status on the association between unhealthy lifestyle factors and adverse health outcomes: a systematic review”. The paper is well-written and there is a careful presentation of the results. However, I have several concerns/comments that I will list, not by order of importance, but by order of appearance. **We thank the reviewer for their positive and helpful comments.**

1. The title mentions “adverse health outcomes” but I think that a better description of what those outcomes are would be more informative. It could read “The influence of socioeconomic status on the association between unhealthy lifestyle factors and cardiovascular disease, cancer and mortality: a systematic review”.

**We thank the reviewer for their suggestion. However, 'adverse health outcomes' is what we use in our protocol, and we justify the choice of selected outcomes (cardiovascular disease, cancer, and mortality) as being the main outcomes linked to 'lifestyle'. Also, it is a helpful phrase that we use a lot in the paper when referring to our outcomes. As a result, we have left the title as it was. **

1. The search was conducted 1.5 years ago and although I am aware of the work involved in running it again I believe that, unless there is a very good reason not to, there should at the very least some mention in the discussion of the number of papers published that met your inclusion criteria in the past 1.5 years.

**We appreciate this suggestion. We have responded to a similar point made by the 1 ^st^ reviewers (Methods, Point 2) by adding the following the strengths and limitations section (lines 600-604): *‘Updating the searches could also identify newer studies, including studies that look at different health outcomes, such as specific cancers and studies from low- or middle-income countries.67,68 It is unlikely that the addition of these studies would change the result around the heterogeneity of data but it could reveal more consistency in terms of SES effects.’***

1. There are other databases that could have been searched as well. I believe the authors should justify why there was a decision not to search them, even if that decision was time constraints and believing that all the studies would be included in PubMed/EMBASE/CINAHL.

**As per our response to the first Reviewers’ Point 1 (Methods) above - We agree that databases such as Scopus and Web of Science may cover additional articles not covered by the databases we selected. However, this project was limited in scope and time and therefore additional databases were felt to be beyond the resources available for this project. We have selected what we felt to be the most relevant databases. And have added the following to the strengths and limitation section (lines 596-600): *‘Our search terms were extensive and the databases we searched likely contained the vast majority of eligible articles 65. Searching additional databases such as Scopus and Web of Science, which are noted for their use as citation indexes rather than primary sources, 66 may have revealed additional eligible studies but this was beyond the time and human resources available for this project.’***

1. Abstract: It would be good to describe briefly which LFs were used or something from this phrase “i. combination of ≥3 LFs: studies that also included metabolic/intermediate factors ( *e.g.*, blood pressure/body mass index (BMI)) as part of their combination of LFs were included so long as the combination also included ≥3 ‘behavioural’ LFs ( *e.g.*, smoking/PA/diet).”

**We thank the reviewer, and we agree this is helpful information for the abstract, which was omitted originally for reasons of word count, but we have added this in now. Similarly, we have also added examples for what we mean by SES in the abstract. The first sentence in the Methods section of the abstract now reads:   ‘Systematic review of studies that examine associations between combinations of >3 LFs *(eg.smoking/physical activity/diet)* and health outcomes and report data on SES *(eg.income/education/poverty-index) *influences on associations.’    **

1. The protocol mentions that all-cause mortality was the main outcome but I didn’t seem to find any mention of it in this paper.

**After running the searches and finding 6 studies that fit our criteria, we felt that there was no need to differentiate by main and secondary outcomes – only 3 studies examined outcomes other than all-cause mortality. Consequently, the largest section of our results (synthesised by outcomes) concerns all-cause mortality so we felt no need to highlight this as the ‘main outcome’ as this is superfluous when few other data exist pertaining to other outcomes.**

1. Introduction: The authors mention different possible explanations but I missed the authors hypothesis of how SES plays moderates the association between lifestyle and health outcomes, or mention which (or a blend of both) of the existing explanations is favoured by the authors.

**We thank the reviewer for this suggestion. However, our objective with this review was to examine whether and in what way SES influences associations between combinations of lifestyle factors and adverse health outcomes. Our aim was not to explore possible mechanisms, and as a group of authors, we are yet to form strong views on which explanatory mechanisms are most likely. The existence of plausible mechanisms for SES effects on these associations are pre-requisite to reviewing the literature for SES effects but understanding the mechanisms and their relative contributions will become much more relevant when the evidence is clear that there are indeed SES effects. Indeed, a review of possible mechanisms would be indicated if clear SES effects were found.   Please see our final sentences in the discussion which we feel respond directly to this comment (lines 624-628):   ‘The aim of this review was to identify and synthesise the evidence for SES modification of associations between LF combinations and adverse health outcomes, not to explain any identified effect modification. However, strong evidence for SES effect modification of such associations could prompt attempts to uncover underlying mechanisms, such as cumulative risks or accelerated biological ageing ^25^^– ^^27^ .’   Relatedly, we have added the following to the introduction to add clarity that we are discussing SES effects on lifestyle associations (line 35):   ‘Beyond differential exposure, further explanations for lifestyle-related health inequalities may involve interactions between LFs and SES; so-called ‘differential vulnerability’ ^17^ , *where SES strengthens the association between lifestyle and adverse health outcomes*.’   And (lines 45-46)   ‘Mechanisms that explain differential lifestyle vulnerability *or that explain how and why SES effects associations between lifestyle and adverse health outcomes* are unclear but could include…’**

1. Figure 2. Most of the HRs are between 1 and 3 but the ticks on the x axis are only every 1-point which doesn’t let me, from the figure alone, see the estimates but only the direction and general magnitude. I suggest adding more ticks and having 1 decimal point instead of 2 (also remember its a log scale). And/or (and ideally) having the HRs and corresponding 95%CI on the plot for every row/analysis. I see that Choi 2020 did not provide confidence intervals or any measure of variability but that should be mentioned in the figure notes.

**We thank the reviewer for these suggestions. As per our response to the first reviewers, we have changed the horizontal axes of Figs 2 & 3 to the log scale. We have also altered the decimal place to 1. The figures are for general visualisation of the data to give an idea of direction and magnitude. However, we have added HRs and CIs to the figures to help the readers see the numerical estimates. We have also made it clear that Choi et al. did not provide CIs.**

1. Supplementary tables should be mentioned along the text when needed .e.g. there is no mention on Table S8 when reporting on the results of the association between combinations of unhealthy LFs and all-cause mortality (figure 2). Also supplementary material are not annexes and table/figures/text should only be included in the paper when these are mentioned.

**We thank the reviewer for this suggestion to add clarity. We have added mentions to the relevant supplementary Tables in the text instead of just referring to the Figures.   In response to the second part of this comment regarding supplementary material and annexes, we are following the journal’s instructions whereby supplementary material is submitted as 'extended data' and published on the publicly available repository of figshare.**

1. Check if abbreviations are defined at their first appearance, .e.g NHANES in the abstract.

**Thank you, this was due to the abstract word limit, but we have added that to the abstract now. We believe all other abbreviations in the manuscript are defined at first mention.**

1. Discussion: There should be some mention about the possible selection bias in the recruitment of these studies, i.e. it is notoriously harder to recruit people from lower SES status

**We agree this is an important caveat that links to all studies relating to SES and have added this to the discussion on study quality (lines 476-478): *‘For example, a limitation to all studies examining SES measures is the difficulty of recruiting participants from less advantaged backgrounds, which introduces selection bias 51,52.’***

1. Discussion: The authors mention how different combinations of LFs were used across studies but these were also measured differently. Please add that besides the difficulty of comparing different combinations of LFs, these were also often times measured in a very different way.

**We thank the reviewer for this point, which we address in our response the 1 ^st^ reviewers who made a very similar point (Discussion point 2). This is the relevant addition we have made to the discussion section (lines 479-487): *‘The heterogeneity and nature of the LF and SES exposure variables examined by the included studies warrants further discussion. Firstly, each LF was measured or surveyed differently (e.g., diet assessed via a national dietary index comprising numerous survey items compared with diet assessed by a few specific food items; SES assess). Secondly, studies varied in their definitions of unhealthy (dichotomisation) for the same LF (e.g., unhealthy alcohol intake defined as >14/>7 weekly drinks for men/women vs. drinking daily or almost daily). Thirdly, dichotomising the LF exposure prior to creating the LF score or combination fails to capture the more complex dose-response and non-linear associations LFs have with adverse health-outcomes 53,54.’***

1. Discussion: Not using individual lifestyle such as smoking or alcohol and instead using lifestyle, combination, etc may have meant that some papers were not picked up. If this was the case or if the authors think this was unlikely, it should be mentioned

**We agree that omitting specific lifestyle factors may have meant some papers were picked up. However, the risk of omission we believe would be lowered by our searching terms such as ‘behaviour’, ‘health factor’, or ‘modifiable factors’ as well as ‘lifestyle’ and ‘combination’ in both the abstract and title. Crucially, our aim was to look at those papers which examined combinations of LFs rather than specific LFs and we feel that our search terms were wide enough to identify papers that looked at combinations of LFs. Further, including some of the specific LFs in the searches has the potential to explode the number of papers identified because the literature on each specific LF is large.**

1. Discussion: “However, the level of evidence from retrospective design is lower” should be taken with caution because it oversimplifies the quality of evidence based on simply the study design. Adding something like “observational retrospective design may, in general, have more biases than a well-designed prospective observational study” would be better

**We thank the reviewer for pointing out this sentence, which, we agree, oversimplifies the issue. The sentence now reads (lines 606-608): *Generally, however, retrospective observational studies may have more biases than well-designed prospective ones*.**

# Reviewer report: Reviewer response for version 1

**DOI:** 10.21956/wellcomeopenres.20745.r54679

**Published:** 2023-04-20

**Authors:** Leandro Garcia, Sophie Jones

**Affiliations:** 1 Centre for Public Health, Queen's University Belfast, Belfast, Northern Ireland, UK

This systematic review aims to identify and appraise the available evidence on whether and how socioeconomic status modifies associations between combinations of lifestyle factors and adverse health outcomes. Overall, the paper is well-written and clearly defines the rationale and objectives for the systematic review. Methods followed the PRISMA guidelines and are described in sufficient detail. The review protocol was registered in PROSPERO. Only six papers met the inclusion criteria, but results are comprehensively and clearly presented, including the tables and figures. The conclusion was limited due to the high heterogeneity of measures for socioeconomic status, a combination of lifestyle factors and mixed findings from included studies. Please find major and minor suggestions below.

 MAJOR POINTS:

*Methods*

1. Comprehensive databases, such as Scopus and Web of Science, that cover potentially relevant journals not covered by the three databases selected, were not used in the search. Have the authors considered the addition of these and/or other databases to enlarge the search coverage? If so, why have other databases not been used?
2. The search was conducted more than one year ago. Do the authors have a good reason to believe that updating the search would not materially change the results and conclusions of the current review? If so, the authors could provide their reasons in the article. Otherwise –and assuming the authors do not have the resources to update the search at the moment- a caveat could be added in the limitations regarding the time elapsed since the search, referencing some of the relevant papers published recently.

*Results*

1. In the discussion, the authors highlight the possibility that different measures of SES (e.g., individual-level vs area-level) may yield different results. Can the authors clarify whether they considered stratifying results to investigate this?
2. Figures 2 and 3: given that different SES indicators were used across papers, and that potentially the modification effect can vary between different SES dimensions, it seems important to indicate in Figures 2 and 3 what SES stratifier was used by each analysis. E.g., presenting the SES stratifier below each author’s name. When possible and relevant, authors can consider similarities and differences between results based on different SES stratifiers.

*Discussion*

1. “However, risk estimates associated with the healthiest and unhealthiest LF combinations where studies share similar LF components (e.g., combination 1: alcohol, unhealthy diet, and physical inactivity vs. combination 2: alcohol, unhealthy diet, physical inactivity, and smoking), are more comparable.”: This statement is accurate only when the additional component(s) are known to have a significantly small contribution to the disease outcome in relation to the contribution of the shared components. In the example given by the authors, the contribution of smoking to mortality is likely to be significantly bigger than any of the other components, maybe significant even when the other components are combined. Hence, risk estimates of quasi-similar combinations cannot be assumed to be comparable in all (or even in most) cases.
2. Many of the lifestyle factors investigated are likely to have a dose-response association with the disease outcomes investigated. Hence, beyond the lack of shared lifestyle factors between studies, the lack of comparable thresholds used to classify (un)healthy behaviour across studies is an important limitation when summarizing the findings. It would be important that the authors acknowledge this, and elaborate on the implications for this and future research.
3. In the introduction, the authors highlight an “An important caveat…” as a justification for the use of the word lifestyle. However, we think there may be something missing about the consistency of the definition of “unhealthy lifestyle” amongst included studies and a discussion about the implication of this on the findings. Could the authors address this somewhere in the discussion or as a limitation?

MINOR POINTS:

1. Aims: in “This systematic review aims to identify, describe, and synthesise the evidence for whether SES modifies associations between combinations of unhealthy LFs and adverse health outcomes”, the authors might consider adding a parenthesis at the end specifying the outcomes investigated, for clarity. E.g., “adverse health outcomes (all-cause mortality, incidence and mortality from CVD or cancer).”
2. PECO criteria: the authors highlight the population as ‘any general adult population’. Could the authors please clarify the age ranges they considered for this population?
3. Data extraction: could the authors please describe how exactly “[point] estimates [and confidence intervals] from studies where the unhealthiest group was the reference were transformed to make the ‘healthiest’ group the reference”?
4. Articles excluded if not in English: we understand that this is common practice in many reviews (albeit PRISMA recommends that articles are not excluded based on language only to avoid biases). However, given that (a) only two papers were not in English, (b) two more papers could be a valuable contribution to the review, given that only six could have been included, (c) these two papers are likely to come from low- or middle-income countries, increasing the diversity of the evidence based analysed, and (d) there are options available to get support with non-English articles (e.g., translation tools, colleagues who speak other languages than English, and Translators without Borders - https://translatorswithoutborders.org/), assessing the eligibility of these two papers based on the PECO criteria and including them in the review if relevant seems like a missed opportunity.
5. In Table 1, for the second to last study presented (Choi *et al.*, 2022), the lifestyle factors for “ii” appear as separate due to an additional return:
  ‘’ii) weekly alcohol intake >14 drinks
  for men, >7 drinks for women …”
  Could the authors check, please?
6. Figures 2 and 3: the x-axis should be in logarithmic scale to ensure visual symmetry between values below and above 1 that indicate the same effect size (e.g., 0.5 and 2).
7. Figure 2: estimates for Choi 2020 do not have the confidence interval. Could author check if it is unintentionally missing?
8. Figure 3: the Eguchi 2017’s analysis for non-CVD, non-cancer mortality seems to be outside the scope of the review, based on its objectives and PECO criteria. The authors should consider removing it from the article.
9. Table S1: some terms seem to be duplicated within searches #2 (see risk reduction   behaviour*[tiab] and *health behaviour[tiab]* with and without asterisks) and #3 (see *health inequalities[tiab]* and social-economic[tiab] with and without asterisk). This does not affect the results of the search but can be corrected for conciseness and clarity. Author might want to check whether the same occurred in Tables S2 and S3.
10. It seems Table S6 is cut short. Could the authors check, please?

OTHER POINTS:

*Background and Discussion*

1. The Background and Discussion sections would greatly benefit from the use of Sydemic Theory to substantiate the rationale for the study and discuss its findings.

Are the rationale for, and objectives of, the Systematic Review clearly stated?

Yes

Is the statistical analysis and its interpretation appropriate?

Not applicable

Are sufficient details of the methods and analysis provided to allow replication by others?

Yes

Are the conclusions drawn adequately supported by the results presented in the review?

Partly

Reviewer Expertise:

Public Health; Physical Activity; Systems Thinking.

We confirm that we have read this submission and believe that we have an appropriate level of expertise to confirm that it is of an acceptable scientific standard, however we have significant reservations, as outlined above.

# Response

**Published:** 2023-11-28

**Authors:** Hamish Foster

**Affiliations:** University of Glasgow, UK

Reviewer comments Authors’ response **1 ^st^ reviewers – Sophie Jones & Leandro Garcia, Centre for Public Health, Queen's University Belfast, Belfast, Northern Ireland, UK**  

 This systematic review aims to identify and appraise the available evidence on whether and how socioeconomic status modifies associations between combinations of lifestyle factors and adverse health outcomes. Overall, the paper is well-written and clearly defines the rationale and objectives for the systematic review. Methods followed the PRISMA guidelines and are described in sufficient detail. The review protocol was registered in PROSPERO. Only six papers met the inclusion criteria, but results are comprehensively and clearly presented, including the tables and figures. The conclusion was limited due to the high heterogeneity of measures for socioeconomic status, a combination of lifestyle factors and mixed findings from included studies. Please find major and minor suggestions below. **We thank the reviewers for their positive comments on the quality of our article.**

**MAJOR POINTS**  

**Methods**  

 1. Comprehensive databases, such as Scopus and Web of Science, that cover potentially relevant journals not covered by the three databases selected, were not used in the search. Have the authors considered the addition of these and/or other databases to enlarge the search coverage? If so, why have other databases not been used? We agree with the reviewers that databases such as Scopus and Web of Science may cover additional articles not covered by the databases we selected. However, this project was limited in scope and time and therefore additional databases were felt to be beyond the resources available for this project. **We have selected what we felt to be the most relevant databases, in discussion with a subject-specific librarian. We have added this as a limitation to the strength and limitations section (lines 596-600):  ‘ *Our search terms were extensive and the databases we searched likely contained the vast majority of eligible articles65. Searching additional databases such as Scopus and Web of Science, which are noted for their use as citation indexes rather than primary sources,66 may have revealed additional eligible studies but this was beyond the time and human resources available for this project.’***

 2. The search was conducted more than one year ago. Do the authors have a good reason to believe that updating the search would not materially change the results and conclusions of the current review? If so, the authors could provide their reasons in the article. Otherwise –and assuming the authors do not have the resources to update the search at the moment- a caveat could be added in the limitations regarding the time elapsed since the search, referencing some of the relevant papers published recently. We thank the reviewers for this point. **We have added the following caveat to the strengths and limitations section (lines 600-604):   ‘ *Updating the searches could also identify newer studies, including studies that look at different health outcomes, such as specific cancers and studies from low- or middle-income countries.67,68 It is unlikely that the addition of these studies would change the result around the heterogeneity of data but it could reveal more consistency in terms of SES effects.***

**Results**  

 1. In the discussion, the authors highlight the possibility that different measures of SES (e.g., individual-level vs area-level) may yield different results. Can the authors clarify whether they considered stratifying results to investigate this? We thank the reviewers for raising this point. As per our protocol, we planned on synthesising data by outcome, but we appreciate that identifying variation in SES effects by SES measure would be valuable. With few included studies we do not believe there is a clear indication for stratifying our results by SES measure but feel this would be an important consideration for future reviews that identify more studies. **We have added the following to the discussion (lines 542-543): ***‘F**uture reviews, with a greater number of included studies, could stratify and synthesise results by SES measure to investigate this further.’***

 2. Figures 2 and 3: given that different SES indicators were used across papers, and that potentially the modification effect can vary between different SES dimensions, it seems important to indicate in Figures 2 and 3 what SES stratifier was used by each analysis. E.g., presenting the SES stratifier below each author’s name. When possible and relevant, authors can consider similarities and differences between results based on different SES stratifiers. **We agree that understanding which SES measure is being used is highly relevant and we thank the reviewer for suggesting adding this data into Figures 2 and 3. We have added the SES measure for each study below the author’s name as well as the corresponding definitions for the most and least advantaged categories (Please see updated Figures 2 and 3). We have updated the legends for these figures accordingly.[HF1]    Without more studies that examine comparable SES measures it is difficult to draw conclusions on how the type of SES measure influences SES effects. For example, of the six studies that examine all-cause mortality, two use income, one uses education, two use area-based measures, and one uses latent class analysis (a combination) with 3 or 4 individual-level SES measures. We have highlighted this in the discussion section (lines 537-541): *‘Having few studies using the same SES measure limited the ability to draw conclusions on how the SES measure influences SES effects. For example, of the six studies that examined all-cause mortality, two use income, one uses education, two use area-based indices, and one uses a combination of income, education level, occupation/employment, and health insurance in a latent class analysis.’***

**Discussion**  

 1. “However, risk estimates associated with the healthiest and unhealthiest LF combinations where studies share similar LF components (e.g., combination 1: alcohol, unhealthy diet, and physical inactivity vs. combination 2: alcohol, unhealthy diet, physical inactivity, and smoking), are more comparable.”: This statement is accurate only when the additional component(s) are known to have a significantly small contribution to the disease outcome in relation to the contribution of the shared components. In the example given by the authors, the contribution of smoking to mortality is likely to be significantly bigger than any of the other components, maybe significant even when the other components are combined. Hence, risk estimates of quasi-similar combinations cannot be assumed to be comparable in all (or even in most) cases. **We thank the reviewer for making this point. We agree that different combinations are not fully comparable for the reason they highlight and the effects of specific combinations remain relative. However, we believe there will be some comparability as unhealthy LFs tend to cluster among individuals.   As a result, we have added the following to the discussion section (Lines 494-526): *‘However, because unhealthy LFs are known to cluster among individuals, participants who report the unhealthiest LF combinations might be comparable even when different LFs are examined 4-8,56. For example, among participants who report the unhealthiest combination of unhealthy LFs in ‘study 1’, there will be some participants with similar numbers and types of unhealthy LFs as those in ‘study 2’ who also report the unhealthiest combination even if study 2 examines fewer LFs because of clustering. This similarity or comparability is more likely where studies share more LF components ( e.g., study 1: alcohol, unhealthy diet, and physical inactivity vs. study 2: alcohol, unhealthy diet, physical inactivity, and smoking). Although, in studies that examine more LFs or have more categories for LF analysis, those classified as the unhealthiest may represent a more extreme group. Nevertheless, *the aim of this review was to identify and appraise all studies that examined the effect of SES on the association between any LF combination and adverse health. Restricting the searches of this review to identify only those studies with the same or similar combinations of LFs would have yielded *even *fewer results *and limited the synthesis. To explore the effect of specific LF combinations and of SES on the associations with adverse health outcomes, future research could attempt to identify the riskiest LF combinations, whether and how the riskiest combinations vary by SES, and whether and how SES effects the associations between specific combinations and adverse health outcomes. Further, there is currently a lack of guidance on how to live in health ways that considers LF combinations and there is no consensus definition for ‘unhealthy lifestyle’ overall. Numerous single LF specific guidelines exist, but these are often too complex to digest for most people and they often fail to account for interactions with other LFs or social contexts 57-59. Therefore, as part of precision medicine, future research could explore the non-linear associations and interactions for a wide range of LFs to define ‘unhealthy’ levels for LFs within specific (or personalised) combinations and across SES the spectrum. These efforts could provide…’***

 2. Many of the lifestyle factors investigated are likely to have a dose-response association with the disease outcomes investigated. Hence, beyond the lack of shared lifestyle factors between studies, the lack of comparable thresholds used to classify (un)healthy behaviour across studies is an important limitation when summarizing the findings. It would be important that the authors acknowledge this, and elaborate on the implications for this and future research. **We thank the reviewers for highlighting this important element of the heterogeneity of LFs examined.   We have expanded on this and its implications both in this study and future research in the discussion section (Lines 479-487):   ‘ *The heterogeneity and nature of the LF and SES exposure variables examined by the included studies warrants further discussion. Firstly, each LF was measured or surveyed differently (e.g., diet assessed via a national dietary index comprising numerous survey items compared with diet assessed by a few specific food items; SES assess). Secondly, studies varied in their definitions of unhealthy (dichotomisation) for the same LF (e.g., unhealthy alcohol intake defined as >14/>7 weekly drinks for men/women vs. drinking daily or almost daily). Thirdly, dichotomising the LF exposure prior to creating the LF score or combination fails to capture the more complex dose-response and non-linear associations LFs have with adverse health-outcomes 53,54. Fourthly,*…’**

 3. In the introduction, the authors highlight an “An important caveat…” as a justification for the use of the word lifestyle. However, we think there may be something missing about the consistency of the definition of “unhealthy lifestyle” amongst included studies and a discussion about the implication of this on the findings. Could the authors address this somewhere in the discussion or as a limitation? **We thank the reviewer for raising this issue and we agree there is a difference between defining what unhealthy levels are for each single LF and defining what an ‘unhealthy lifestyle overall’ might be when considering a combination of LFs.   In the included studies, different numbers of categories were used for examining the associations with a combination of LFs (and then the effect of SES on those associations). The number of categories chosen was partly dependent on the number of LFs included (e.g., Eguchi 2017 et al. included 8 LFs but examined the associations between mortality and 5 lifestyle categories where participants were scored 0–3, 4, 5, 6, 7–8 based on how many LFs they had). However, the included studies did not actually define what ‘unhealthy lifestyle’ overall was, but rather, in our review, we extracted the estimates for the most extreme lifestyle categories (healthiest and unhealthiest) and we labelled these as ‘healthy’ and ‘unhealthy’, respectively. We appreciate this has implications for our main comparator where we selected estimates for the healthiest vs unhealthiest lifestyle overall in both the most and least advantaged SES groups. As a result, we have added the following to the limitations section (Lines 613-624): *‘Our decision to extract the ‘healthiest’ vs. ‘unhealthiest’ in both the most and least advantaged groups as our main comparator may have limited our synthesis. Examining the effect of SES on associations between the extremes of lifestyle and adverse health outcomes may miss how SES might affect the relationships in more nuanced ways as it relies on the assumption that SES effects will be seen at the extremes of lifestyle. However, we also extracted results for interactions between SES and LFs, which provided further evidence for whether and how SES effects associations between LFs and adverse health outcomes. As more studies examine associations between combinations of LFs and adverse health outcomes in more detail (e.g., by examining non-linear associations and using continuous rather than categorical or ordinal variables for LF combinations) 53,54, future reviews could examine how SES effects the shape of relationships between LFs and adverse health outcomes.’*   As well as the above, we also wonder if there might be a lack of clarity here on our part. The section in the introduction starting, ‘An important caveat…’, may have been misleading. We only mean to highlight the word ‘lifestyle’ rather than the concept of ‘unhealthy lifestyle overall’. This is because the negative connotations associated with the word lifestyle explained in the manuscript apply equally to single LFs or combinations of LFs (i.e., ‘unhealthy lifestyle overall’). The word lifestyle is the problem and therefore we have removed ‘unhealthy’ from the relevant paragraph in the introduction to make this clearer. And, in view of the reviewers’ point about defining ‘unhealthy lifestyle’ overall, we have added the following to the discussion (lines 517-521): *‘Further, there is currently a lack of guidance on how to live in health ways that considers LF combinations and there is no consensus definition for ‘unhealthy lifestyle’ overall. Numerous single LF specific guidelines exist, but these are often too complex to digest for most people and they often fail to account for interactions with other LFs or social contexts 57-59.’***

**MINOR POINTS**  

1. Aims: in “This systematic review aims to identify, describe, and synthesise the evidence for whether SES modifies associations between combinations of unhealthy LFs and adverse health outcomes”, the authors might consider adding a parenthesis at the end specifying the outcomes investigated, for clarity. E.g., “adverse health outcomes (all-cause mortality, incidence and mortality from CVD or cancer).”

**Thank you. We have added this suggestion.**

1. PECO criteria: the authors highlight the population as ‘any general adult population’. Could the authors please clarify the age ranges they considered for this population?

**We have added the following to our PECOS criteria:   ‘1) Population: any general adult population *(age ≥18 years)*…’**

1. Data extraction: could the authors please describe how exactly “[point] estimates [and confidence intervals] from studies where the unhealthiest group was the reference were transformed to make the ‘healthiest’ group the reference”?

**Thank you. We have added the following the relevant data extraction section (lines 135-140): *‘This transformation was achieved by dividing: 1) all hazard ratios (HRs) by the HR of the healthiest category (the healthiest category HR then becomes 1.00), 2) all lower confidence intervals (CIs) by the lower CI of the healthiest category, and 3) all upper CIs by the upper CI of the healthiest category. This then requires swapping the upper and lower CIs because transformed lower CIs become upper CIs.’***

1. Articles excluded if not in English: we understand that this is common practice in many reviews (albeit PRISMA recommends that articles are not excluded based on language only to avoid biases). However, given that (a) only two papers were not in English, (b) two more papers could be a valuable contribution to the review, given that only six could have been included, (c) these two papers are likely to come from low- or middle-income countries, increasing the diversity of the evidence based analysed, and (d) there are options available to get support with non-English articles (e.g., translation tools, colleagues who speak other languages than English, and Translators without Borders - https://translatorswithoutborders.org/), assessing the eligibility of these two papers based on the PECO criteria and including them in the review if relevant seems like a missed opportunity.

**We thank the reviewer for their suggestion of arranging translations for these 2 papers. We were not aware of freely available high quality translation tools but this is certainly something we can use in the future. We agree that adding more diverse studies would be of benefit. However, unfortunately, we currently do not have the human resources to investigate free and quality translation tools to then translate, review, and or synthesise these studies into our review.**

1. In Table 1, for the second to last study presented (Choi *et al.*, 2022), the lifestyle factors for “ii” appear as separate due to an additional return:
  ‘’ii) weekly alcohol intake >14 drinks
  for men, >7 drinks for women …”
  Could the authors check, please?

**Thank you. This was a formatting error, which we have corrected.**

1. Figures 2 and 3: the x-axis should be in logarithmic scale to ensure visual symmetry between values below and above 1 that indicate the same effect size (e.g., 0.5 and 2).

**We thank the reviewer for this suggestion. We have altered the x-axes to logarithmic scales. We hope this makes them more interpretable for readers.**

1. Figure 2: estimates for Choi 2020 do not have the confidence interval. Could author check if it is unintentionally missing?

**This is correct. Unfortunately, confidence intervals are not provided for all the results in their manuscript. Instead, whether or not it was significant (p<0.05) was indicated.  **

1. Figure 3: the Eguchi 2017’s analysis for non-CVD, non-cancer mortality seems to be outside the scope of the review, based on its objectives and PECO criteria. The authors should consider removing it from the article.

**The reviewer is correct, and we have removed that data and corresponding results from the figures and manuscript. We have made this clear in the results section (lines 175-176): *‘The non-CVD and non-cancer results are not reported here as they are outside the scope of this review.’***

1. Table S1: some terms seem to be duplicated within searches #2 (see risk reduction   behaviour*[tiab] and *health behaviour[tiab]* with and without asterisks) and #3 (see *health inequalities[tiab]* and social-economic[tiab] with and without asterisk). This does not affect the results of the search but can be corrected for conciseness and clarity. Author might want to check whether the same occurred in Tables S2 and S3.

**We thank the reviewer for pointing this out. There was an error because of autocorrect to UK English. We had searched using both American and UK English spellings of the word ‘behaviour’ (behavior). We have now corrected #1 in Table S1 to show that. With this correction, there is no duplication with both UK and American English spelling searched for in MeSH terms and in title/abstracts separately. We do not see any duplication in #3. We have also checked Table S2 and S3 and there is no duplication.**

1. It seems Table S6 is cut short. Could the authors check, please?

**We have checked the original downloadable version from figshare and Table S6 is complete but it does go over 2 pages. Perhaps this was causing an issue for the reviewer but it appears correct when we have downloaded a new version as a reviewer or reader would. We have also ensured the new corrected version uploaded onto figshare is also complete. OTHER POINTS: Background and Discussion The Background and Discussion sections would greatly benefit from the use of Sydemic Theory to substantiate the rationale for the study and discuss its findings. We thank the reviewer for their suggestion. We agree Syndemic Theory could be a helpful theoretical tool to examine synergy and additive interactions between LFs and SES. However, applying Syndemic Theory to our study somewhat presupposes the presence of synergistic or additive interactions between LFs and SES. Whereas our aim was to examine for the presence of these interactions. If evidence for LF-SES interactions is strengthened then Syndemic Theory could be a useful theoretical lens with which to examine and or explain those interactions.**

**[HF1]Check**
